# Enhancing insights: exploring the information content of calorespirometric ratio in dynamic soil microbial growth processes through calorimetry

**DOI:** 10.3389/fmicb.2024.1321059

**Published:** 2024-02-02

**Authors:** Shiyue Yang, Eliana Di Lodovico, Alina Rupp, Hauke Harms, Christian Fricke, Anja Miltner, Matthias Kästner, Thomas Maskow

**Affiliations:** ^1^Helmholtz Centre for Environmental Research – UFZ, Leipzig, Germany; ^2^Rheinland-Pfälzische Technische Universität Kaiserslautern-Landau (RPTU), Landau in der Pfalz, Germany

**Keywords:** calorimetry, biothermodynamics, energy use efficiency, carbon use efficiency, growth rate, calorespirometric ratio, soil systems

## Abstract

Catalytic activity of microbial communities maintains the services and functions of soils. Microbial communities require energy and carbon for microbial growth, which they obtain by transforming organic matter (OM), oxidizing a fraction of it and transferring the electrons to various terminal acceptors. Quantifying the relations between matter and energy fluxes is possible when key parameters such as reaction enthalpy (*∆*_r_*H*), energy use efficiency (related to enthalpy) (EUE), carbon use efficiency (CUE), calorespirometric ratio (CR), carbon dioxide evolution rate (CER), and the apparent specific growth rate ( $\mu_{\mathrm{app}}$ ) are known. However, the determination of these parameters suffers from unsatisfying accuracy at the technical (sample size, instrument sensitivity), experimental (sample aeration) and data processing levels thus affecting the precise quantification of relationships between carbon and energy fluxes. To address these questions under controlled conditions, we analyzed microbial turnover processes in a model soil amended using a readily metabolizable substrate (glucose) and three commercial isothermal microcalorimeters (MC-Cal/100P, TAM Air and TAM III) with different sample sizes meaning varying volume-related thermal detection limits (*LOD*_*v*_) (0.05 −1 mW L^−1^). We conducted aeration experiments (aerated and un-aerated calorimetric ampoules) to investigate the influence of oxygen limitation and thermal perturbation on the measurement signal. We monitored the CER by measuring the additional heat caused by CO_2_ absorption using a NaOH solution acting as a CO_2_ trap. The range of errors associated with the calorimetrically derived $\mu_{\mathrm{app}}$ , EUE, and CR was determined and compared with the requirements for quantifying CUE and the degree of anaerobicity ( $\eta_{A})$ . Calorimetrically derived $\mu_{\mathrm{app}}$ and EUE were independent of the instrument used. However, instruments with a low *LOD_v_* yielded the most accurate results. Opening and closing the ampoules for oxygen and CO_2_ exchange did not significantly affect metabolic heats. However, regular opening during calorimetrically derived CER measurements caused significant measuring errors due to strong thermal perturbation of the measurement signal. Comparisons between experimentally determined CR, CUE, $\;\eta_{A}$ , and modeling indicate that the evaluation of CR should be performed with caution.

## 1 Introduction

Calorimetry is a non-destructive technique that was initially used to measure the heat released by small rodents (Crawford, 1788; Lavoisier and DeLaplace, 1994). The results sparked scientists’ interest, leading to the development of more sensitive and high-throughput calorimeters (Sunner and Wadsö, 1959; Hofelich et al., 2001; Wadsö et al., 2010; Paufler et al., 2013; Wadso, 2015; Wadsö et al., 2017) for application to various life-forms, such as animals and plants (Kemp and Guan, 1997), microbes (Gustafsson, 1991; von Stockar et al., 1993; Fricke et al., 2019), as well as entire soil systems (Dijkerman, 1974; Ljungholm et al., 1979; Herrmann and Bölscher, 2015).

Soil plays an essential role in maintaining the Earth’s carbon balance. This balance heavily depends on catalytic functions performed by microbial communities on soil organic matter (SOM), whose activity obeys the rules of thermodynamics for an open system. A crucial state function for testing and evaluating thermodynamic models is the reaction enthalpy $\Delta_{r}H$ , which can be determined by isothermal microcalorimetry. The measured heat production rate P (in W) contains both kinetic and stoichiometric information, and the integral of the heat production rate provides (under constant pressure) the reaction enthalpy ( $\Delta_{r}H=Q$ ).

In soil systems, organic matter (OM) can be utilized for growth through microbial assimilation in anabolic reactions or dissipated as CO_2_ to the environment in catabolic reactions. The catabolic reactions provide the energy for the anabolic reactions (Kästner et al., 2021) and essentially determine the overall heat production rate of the overall reactions (Canfield et al., 2005; von Stockar, 2010). This explains the frequently observed relation between the CR and the yield coefficient in biotechnology or the CUE in soil science (Von Stockar and Birou, 1989; Hansen et al., 2004; Herrmann and Bölscher, 2015; Wadsö and Hansen, 2015).

A simple calorimetric experiment can provide many important and valuable thermodynamic, kinetic and stochiometric variables (a detailed derivation is given in section 2.4). For example, *P* is equal to the product of the growth rate multiplied by the reaction enthalpy. The slope of the natural logarithm of *P* vs. time corresponds to the apparent growth rate $\left(\mu_{\mathrm{app}}\right)$ . In the simplest case, the slope is constant over a certain time. $\Delta_{r}H$ is linked to the growth reaction stoichiometry via the law of Hess (Braissant et al., 2010; Maskow and Paufler, 2015). Applying the law of Hess requires both calorimetric information and information about matter fluxes. To link the two pieces of information, a combination of calorimetry and respirometry, also known as calorespirometry is required. From such coupled measurements, the calorespirometric ratio (CR) is obtained, which represents the ratio of the specific heat production rate *P_m_* (in W g^−1^) to the specific CO_2_ evolution rate CER (in mol g^−1^ s^−1^) or the ratio of the specific total heat *Q_m_* (in J g^−1^) to the specific total evolved CO_2_ (in mol g^−1^). Thus, the CR has the dimension of J mol^−1^.

**Figure 1:**
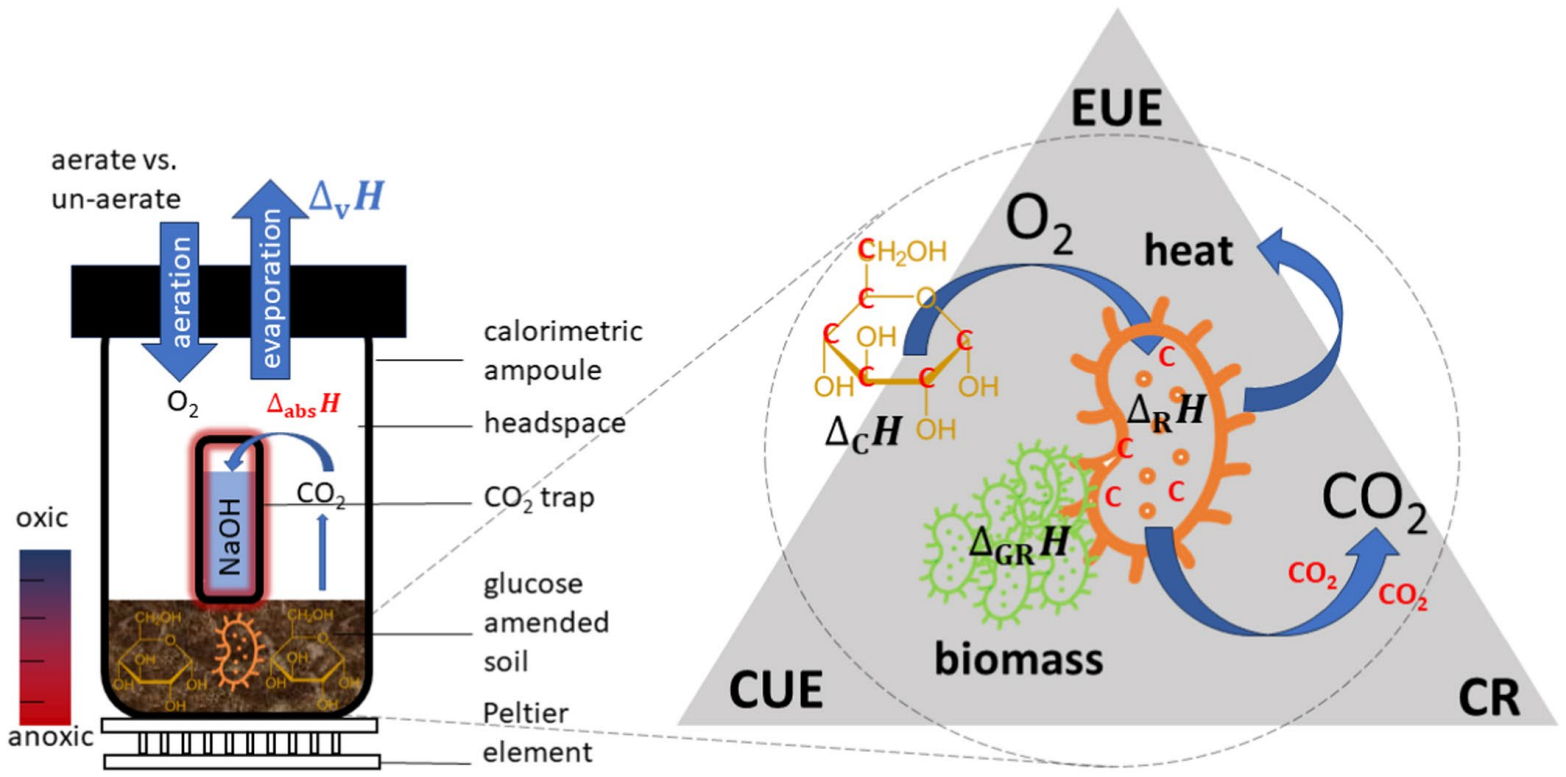
Setup of a common calorespirometric measuring system to monitor simultaneously *P* and CER **(left)**. Fundamental mechanism for microbial turnover of substrates in soil and underlying key variables for evaluating the efficiency of such processes **(right)**.

Three options of measuring CR have recently been discussed. Firstly, the heat production rate of soil samples with (*P*_SN_) and without (*P*_S_) a CO_2_ trap (filled with a trap solution, usually NaOH) can be continuously monitored calorimetrically. A simple setup is shown in Figure 1. Additional heat is released by the absorption reaction between CO_2_ and NaOH (2NaOH + CO_2_ → Na_2_CO_3_ + H_2_O) causing an increase in *P*_SN_ compared to *P*_S_ in vials without NaOH trap. The CER is calculated from the difference between both signals and the known reaction enthalpy for the CO_2_ trapping reaction ( $\Delta_{\mathrm{abs}}H)$ (Critter et al., 2001; Barros et al., 2010). Secondly, like the first approach, a CO_2_ trap can be positioned in a calorimetric ampoule or a backup reactor, and the trap can be sampled at defined time points and the trapped CO_2_ quantified, for example by titration or a dissolved inorganic carbon analyzer. In all cases, the total inorganic carbon in the trap is a measure of the CER (Barros et al., 2011). Thirdly, the heat production rate of soil samples can be monitored in a calorimeter, and the headspace of the calorimetric ampoules or of parallel back-up reactors can be sampled at defined time points and quantified by alternative analytics, such as gas-chromatography, as discussed in Pushp et al., 2021.

To link calorespirometric data and energy and matter turnover in soil systems, thermodynamic models are being developed to investigate the link between CR, CUE and EUE. Hansen et al. (2004) established a quantitative model linking CR with CUE, which is widely used today. To achieve this connection, Hansen et al. (2004) simplified the intricate soil processes by assuming aerobic metabolisms, concentrating on a single substrate, and disregarding interactions of OM with minerals. Furthermore, Chakrawal et al. (2020) extended Hansen’s model to encompass anaerobic, fermentative processes that result in the production of ethanol and lactic acid. This expansion allows for a more comprehensive understanding of the relationship between CR and CUE, considering a broader range of metabolic pathways and conditions. This is particularly important in complex, heterogeneous and dynamic systems such as soil. Other models were established to predict the fate of carbon (Trapp et al., 2018) as well as the conversion rates of SOM (Ugalde-Salas et al., 2020).

However, based on the overall turnover reactions, energy and mass balances depend on the microbial growth, decaying of cells, CO_2_ formation and transformation from necromass to SOM (Kästner et al., 2021). Deriving energy turnover parameters in soil samples using isothermal microcalorimeters (IMCs) and applying them to thermodynamic models is still challenging for the following reasons. First, 1 g of soil can contain approximately 10^9^ microbial cells from 4,000 different microbial taxa (Raynaud and Nunan, 2014; Chaudhary et al., 2019). However, a large fraction of them is in a dormant state at any given time (Blagodatsky and Richter, 1998), with a metabolism limited to basic maintenance of the cells. The heat output of these resting soil microbes is so low that highly sensitive calorimeters are required, or the sample sizes increased to make it measurable. Second, natural spatial heterogeneities and complexity in different soils influence the accuracy of the heat signal. This influence can be reduced by measuring large soil samples. The combined effect of the minimal thermal limit of detection and maximal sample size can be considered by comparing the minimum volume-related declared thermal limit of detection (*LOD_V_*) of different devices, Third, oxygen depletion triggered by microbial activities might results in anaerobic conditions in the sample ampoules. Thus, during the measurements, it is common to open the calorimetric ampoules from time to time, not only to sample the NaOH solution or to renew the CO_2_ trap but also to counteract the consumption of oxygen to avoid anaerobic conditions (see Figure 1). Aeration becomes important when the experiments run for long periods (e.g., weeks or months) or the microorganisms are highly active. However, a weekness of opening the ampoules is the thermal disturbance caused by a sudden temperature change when removing and replacing the ampoule on the Peltier sensor. This is particularly important for signals in the microwatt range (Wadsö, 2001).

A few aspects need to be considered to prevent over- and mis-interpretation of the calorimetric signals in soil research: (i) how accurately can the CR be determined in the best case using commercially available IMCs, (ii) what influences do the *LOD_V_* and the regular opening of the calorimetric measuring chamber have on the determination of kinetic ( $\mu_{\mathrm{app}}$ ) and thermodynamic parameters ( $\Delta_{r}H\;$ , EUE, CR), (iii) do rates or integrated values give the most reliable CR, and (iv) what are the consequences of the experimental error of the CR determination for the calculation of CUE and the degree of anaerobicity ( $\eta_{A}$ ) are?

This study thus aims at maximizing the achievable information about energy turnover from calorimetric experiments with soil samples. For this purpose, substrate-induced growth experiments on soil samples were performed with glucose as a readily metabolizable substrate, which is expected to rapidly give clear calorespirometric signals. The experiments were conducted with different instruments with soil treated in the same way. We tested different ways to analyze the data. Theoretical expectation on the relation between the CR and the CUE or the $\eta_{A}$ and minimum requirements for CR accuracy determination are also discussed.

## 2 Materials and methods

### 2.1 Technical and preparative framework

The three different types of commercial available IMCs provide the technical framework for this study, and are intended to evaluate the combined effects of sample sizes and thermal detection limits on both, the calorimetric signal itself and the derived values (e.g., CUE, CER, EUE, $\mu_{\mathrm{app}}$ , CR). For an optimal performance of the IMCs, two factors are crucial: high thermal sensitivity and large soil sample size. This is expressed by the *LOD_V_*. Further technical details regarding these instruments are provided in Table 1, with references to the respective sources (operational manuals from manufacturers).

**Table 1:** Technical comparison of the calorimeters used (LOD-thermal limit of detection, *LOD_*V*_* minimum thermal limit of detection).

Instrument	Maximum number of channels	*LOD* μW	Declared signal drift over 24 h μW	Volume of the reaction vessel mL	*LOD_*V*_* mW L^−1^
MC-Cal/100P	12	20	<40	20	1
TAM Air	8	4	<40	20	0.2
TAM III	24	0.2	<0.2	4	0.05

In order to avoid unknown heat losses to the environment or to minimize the impact of water evaporation, calorimetric measurements are conducted in air-tight closed ampoules (Figure 1). However, due to the rapid depletion of oxygen by active microbial communities within the closed ampoules, oxygen might get limited, shifting the metabolism toward anaerobic processes, with lower Δ_r_*H*. A potential limitation by insufficient oxygen supply can be mitigated by periodically aerating the ampoules. To quantify the impact of regularly aerating the ampoules, two types of experiments were performed: one with aeration (indexed as A) and another permanently un-aerated (indexed as U) using the calorimeter with the lowest *LOD_V_* (TAM III). Oxygen limitation can simply be estimated assuming ideal gas behavior as described in Eq. 1.


(1) $$n_{O_{2}}=\frac{p\cdot V}{\chi_{O_{2},air}\cdot R\cdot T}$$

Here, *p*, *V*, $\;\chi_{O_{2},air}$ , *R*, *T* stand for the pressure (101,325 Pa), the gas volume of the ampoule, the mole fraction of oxygen in the air (0.2094) (Lemmon et al., 2000), the universal gas constant (8.314 J mol^−1^ K^−1^) and the temperature in K, respectively. The maximum required oxygen can be estimated assuming a complete oxidation of glucose (see Eq. 2).


(2) $$C_{6}H_{12}O_{6}+6O_{2}\to 6{\mathrm{CO}}_{2}+6H_{2}O$$

We added 900 μg (0.005 mmol) glucose or 360 μg (0.03 mmol) C per g dw-soil. Thus, we expect a maximum oxygen consumption of 0.03 mmol g^−1^ dw-soil. For the estimation of oxygen availability, the compact volume of the soil needs to be considered as soil is a porous structure. Thus, oxygen within soil particles pores should also be considered. The density of the dry soil particles is estimated to be 2.65 $g\;{\mathrm{cm}}^{-3}$ (Schjønning et al., 2017). Table 2 compares the available amount of oxygen with the required amount of oxygen as well as the maximum expected CO_2_ concentration in the headspace. The calculations are provided in the Supplementary material. The estimation indicates that oxygen consumption may be significant, and oxygen limitation might become an issue if all glucose is respired.

**Table 2:** Summary of the oxygen availability and maximum expected CO_2_ concentration in all calorimeters used.

Calorimeter	Volume of the ampoule ml	Available air volume ml	Estimated O_2_ availability mmol	Maximum required O_2_ mmol	Maximum CO_2_ concentration %
MC-Cal/100 P	20	17.92	0.16	0.12	15.0
TAM Air	20	17.92	0.16	0.12	15.0
TAM III	4	3.58	0.03	0.02	15.0

### 2.2 Soil preparation

As an example, farmyard manure soil from the Dikopshof long-term experiment (since 1904) from INRES (Institute of Crop Science and Resource Conservation), Bonn University was used in these experiments. The soil is classified as Haplic Luvisol (Parabraunerde), with a silt loam texture, pH 6.3, 0.74% organic carbon and a water holding capacity of 31% (w dw^−1^ soil). The soil has been treated with farmyard manure fertilizer annually and it is aggregated moderately with a moderate usable field capacity. Further physicochemical parameters of the soil are well-documented and can be found here https://www.lap.uni-bonn.de/en/research/projects/long-term-experiment-dikopshof (Huging, 1904).

In order to prepare the soil samples, they were air-dried and stored at room temperature (approximately 20°C). The air-dried soil was initially sieved through a 2-mm sieve, with larger aggregates crushed and stones removed. The sieved soil was then transferred into a glass beaker. Roots, seeds, and other organic material were carefully taken out. To obtain around 14% (w dw^−1^ soil) water content for the pre-incubation, deionized water was added stepwise to the dried soil and manually stirred for homogeneous distribution. A smaller glass beaker, partially filled with water, was placed on the soil surface to maintain the moisture. The larger glass beaker was sealed with parafilm and pre-incubated for 7 days at 20°C.

### 2.3 Calorimetric soil incubation experiments

In the following study, the IMCs TAM III (Minicalorimeter/Multi 4 mL), TAM Air (TA Instruments, New Castle, USA) equipped with 12 and 8 channels, respectively, as well as the MC-Cal/100P (C3 Prozess- und Analysetechnik GmbH, Munich, Germany) equipped with 12 measuring channels and 2 reference channels were used to perform the substrate-induced soil experiments. The three calorimeters differ in a few main characteristics, which can be found in Table 1. After the pre-incubation period, a glucose solution (200 g L^−1^) was added to the pre-incubated soil using a pipette aiming for a concentration of 360 μg C g^−1^ DW soil, which corresponds to four times the microbial carbon content quantified via chloroform fumigation extraction. With the addition of the glucose solution, a water content of 16% was reached. In control samples, 16% water content was reached via the addition of deionized water. The entire calorimetric incubations were performed at 20°C.

In our first research question, we would like to investigate what influences the *LOD_*V*_* of different IMCs have on the determination of kinetic ( $\mu_{app}$ ) and thermodynamic parameters. Soil was prepared following the same method mentioned previously. Afterwards, the glucose amended soil (min. 99%, CHEMSOLUTE) and unamended soil were distributed equally into the different calorimetric glass ampoules in order to make full use of all channels in each device, various numbers of replicates were used in different IMCs, which can be found in Table 3. We aimed at providing sufficient oxygen for aerobic microbial activity while having sufficient soil to obtain a good heat signal. Moreover, we maintained the air/soil volume ratio in all experiments at 4.44. Therefore, 4.5 g of wet soil (16% water content) were used for 20 mL glass ampoules (TAM Air and MC-Cal/100 P), while 0.9 g of wet soil (16% water content) were used for 4 mL glass ampoules (TAM III).

**Table 3:** Experimental set-up.

Abbreviation	Set-up	Influence of *LOD_v_*			Influence of aeration
		MC-Cal/100P	TAM Air	TAM III	TAM III
*P*_S_(*t*)	Soil, un-aerated	(*n* = 3)	(*n* = 4)	(*n* = 6)	(*n* = 2)
*P*_SN_(*t*)	Soil, NaOH, un-aerated	/	/	/	/
*P*_SG_(*t*)	Soil, Glucose, un-aerated	(*n* = 3)	(*n* = 4)	(*n* = 6)	(*n* = 2)
*P*_SGA_(*t*)	Soil, Glucose, reg. aerated	/	/	/	(*n* = 2)
*P*_SGNU_(t)	Soil, Glucose, NaOH, un-aerated	/	/	/	(*n* = 3)
*P*_SGNA_(*t*)	Soil, Glucose, NaOH, reg. aerated	/	/	/	(*n* = 3)

Secondly, to figure out the influence of aeration on the heat production rate ( $P_{m}$ ) and metabolic heat ( $Q_{m}$ ) measurement and CER calculation, aeration experiment was conducted in TAM III. Aeration could have huge thermal disturbance on calorimetric signals. Therefore, IMCs with most stable temperature control was utilized to conduct experiments and answer this research question to avoid misinterpretation of the result. Triplicates were used for each treatment. At each aeration time (*t* = 8, 22, 30, 50 h), calorimetric ampoules were taken out from TAM III to prevent oxygen depletion. NaOH containers were also moved and NaOH solution was renewed to avoid saturation. Lids were opened and ampoules were left open for aeration under 20°C room temperature for 5 min. Afterwards, the ampoules were closed tightly and introduced again into the original channels. Half of the ampoules contained a small vial with 700 μL, 0.4 M NaOH ( ≥98% , Carl Roth GmbH) to measure the combined heat of metabolism and absorption of CO_2_.

In the case of the TAM III instrument, the prepared ampoules were placed into the channels of the calorimeter and allowed to thermally equilibrate for 15 min in the pre-heating position. After another 45 min for thermal equilibration in the measuring position, *P* was recorded. Regular gain calibration was performed to ensure the measurement precision. This involves generating heat pulses in each channel using an integrated electrical calibration heater (Joule heat). The resulting calibration data provided gain factors and offsets for each channel, which were applied by the instrument. The TAM III instrument has fixed installed reference directly below the measurement channel. For TAM Air, the baseline automatically started and was recorded for 30 min (time needed to have a stable signal according to the experimental wizard’s criteria). Afterwards, both measuring and reference ampoules were placed directly in the measuring position. Heat production rate recording commenced after 45 min when the data were considered correct by the software (thermal equilibration). As with the TAM III, calibration resulted in gain factors and offsets for each channel, ensuring accurate measurements. For the MC-Cal/100P instrument, an internal electrical calibration was performed before conducting the experiments. The instrument automatically determined and applied gain factors and offsets of each channel. The prepared ampoules were directly placed in the measuring position, which required a longer time (60 min) until the instrument provided stable data. One channel per block was selected as a reference and contained a reference ampoule.

The reference ampoules for TAM Air and MC-Cal/100P were filled with 1.362 mL deionized water to give a heat capacity similar to the soil samples. All measurements were conducted at 20°C. We stopped all experiments when all calorimetric signals were constant over time. However, for comparison, we evaluated the signals until 70 h.

Table 3 summarizes the different set-ups in the respective calorimeters and the number of replicated used (n); note that not all the set-ups were replicated in all the devices.

### 2.4 Theoretical framework

Based on the experimental data that was obtained from the calorespirometric measurements a theoretical framework can be developed to derive important and valuable thermodynamic and kinetic parameters of soil microbial processes. The heat production rate *P*(*t*) is linked to the rate *r_i_* (*t*) of all *i* occurring reactions and their respective reaction enthalpies $\Delta_{r}H_{i}$ using Eq. 3 (Assael et al., 2023).


(3) $$P\left(t\right)=\overset{n}{\underset{i=1}{\sum }}r_{i}\left(t\right)\cdot \Delta_{r}H_{i}\;$$

The total heat, *Q*(*t*), results from the integration of the heat production rate, as given in Eq. 4.


(4) $$Q\left(t\right)=\overset{t}{\underset{t=0}{\int }}P\left(t\right)dt$$

Performing integration to the end of the reaction and dividing the total heat by the amount of substrate consumed yields the reaction enthalpy $\Delta_{r}H$ , which contains stoichiometric information (Eq. 5). It is typically assumed that glucose is a rapidly and almost completely degraded substrate (Yang et al., 2016). Therefore, $n^{e}$ is assumed in this work to be 0 after 70 h.


(5) $$\Delta_{r}H=\frac{\int_{t_{0}}^{t^{e}}P\left(t\right)dt}{n_{0}-n^{e}}=-\overset{n}{\underset{i=1}{\sum }}Y_{i/S}\cdot \Delta_{C}H_{i}$$

Here, *t*_0_, *t*^e^*, n_0_, n*^e^*, Y_i/S,_* $\Delta_{C}H_{i}$ stand for the time of the beginning and end of the metabolic reaction, the amount of the substrate before and after the reaction, the yield coefficient, and the combustion enthalpy of the compound *i*, respectively. The yield coefficient expresses the amount of the component *i* required or formed during the conversion of one mol consumed substrate. In soil sciences, energy use efficiency (EUE) is an important parameter which can be defined in different ways. In the following, we will define EUE as shown by Eq. 6.


(6) $$\mathrm{EUE}=1-\frac{Q}{\left(n_{0}-n^{e}\right)\cdot \Delta_{c}H_{Glucose}}$$

Here, *Q*, *n*_0_, $n^{e}$ , $\Delta_{C}H_{Glucose}$ stand for the measured total heat over the whole reaction, the amount of added glucose, the amount of glucose after the reaction, and the combustion enthalpy of glucose, respectively.

In the simplest case of a pure microbial culture, when putting all metabolic reactions together and assuming exponential growth after the addition of the substrate, an exponential curve with an apparent specific growth rate $\mu_{\mathrm{app}}$ is expected and indeed, this is mostly observed after adding a C- and energy source (Eq. 7). In the case of soil samples, we observe an exponential growth phase, but it is the results of overlapping metabolisms, due to the complexity of the soil system. Sometimes a lag phase is observed which for simplicity, is not reflected in the following equation. If a lag phase is present, it would mainly affect the timing, but not the slope of the curve of ln( P ( t )) vs. t .


(7) $$P\left(t\right)=P_{0}\cdot \exp\left(\mu_{\mathrm{app}}\cdot t\right)$$

This means that plotting the ln(*P*(*t*)) vs. *t* gives a straight line with the slope of the apparent specific growth rate $\mu_{\mathrm{app}}$ (Figure 1). In order to capture the metabolic heat production rate, *P*(*t*), after substrate addition, both heat production rates of soil amended with glucose (SG) ( $P_{\mathrm{SG}}(t)$ ), and unamended soil(S), ( $P_{S}(t)$ ) must be measured and the metabolic heat production rate of substrate metabolization is the difference between $\left(P_{\mathrm{SG}}(t\right)$ ) and $P_{S}\left(t\right)$ (Eq. 8).


(8) $$P\left(t\right)=P_{\mathrm{SG}}\left(t\right)-P_{S}\left(t\right)$$

An important parameter in thermodynamic soil research is the calorespirometric ratio (CR), which correlates the released heat with the evolved carbon dioxide. It can be defined from the *P* and CER (CR*_P_*, Eq. 9), or from *Q* and the accumulated amount of released CO_2_, (CR*_Q_*, Eq. 10). Both approaches were tested and discussed in the respective sections.


(9) $${\mathrm{CR}}_{P}=\frac{P\left(t\right)}{\mathrm{CER}\left(t\right)}$$

(10) $${\mathrm{CR}}_{Q}=\frac{Q\left(t\right)}{\int_{t=0}^{t}\mathrm{CER}\left(\tilde{t}\right)\;d\tilde{t}}\;$$

The CR is important since under aerobic conditions it is thought to contain information about the CUE (Hansen et al., 2004; Maskow et al., 2011). Additionally, newer modeling research revealed that the CR contains also information about the ratio of aerobic to anaerobic metabolisms (Chakrawal et al., 2020). However, for calculation of the CR, the CER needs to be measured, which is often done by equipping a calorimetric ampoule with a CO_2_ trap (NaOH solution, subscript N) and monitoring the additional heat of the CO_2_ absorption reaction ( $\Delta_{\mathrm{abs}}H=108.4\;\mathrm{kJ}\;{\mathrm{mol}}^{-1})$ (Criddle et al., 1991). $P_{\mathrm{SN}}$ is the heat production rate of unamended soil equipped with CO_2_ trap and $P_{S}$ is the heat production rate of unamended soil. $P_{\mathrm{SGN}}$ is the heat production rate of glucose-amended soil equipped with CO_2_ trap and $P_{\mathrm{SG}}$ is the heat production rate of glucose-amended soil. In the case of unamended (S) and glucose amended (SG) soil, the CER can be calculated according to Eqs. 11, 12.


(11) $$CER_{S}\left(t\right)=\frac{P_{\mathrm{SN}}\left(t\right)-P_{S}\left(t\right)}{\Delta_{\mathrm{abs}}H}\;$$

(12) $$CER_{\mathrm{SG}}\left(t\right)=\frac{P_{\mathrm{SGN}}\left(t\right)-P_{\mathrm{SG}}\left(t\right)}{\Delta_{\mathrm{abs}}H}$$

### 2.5 Statistical analysis

The statistical analysis (Kruskal-Wallis test for ≥2 nonparametric groups and Wilcoxon test for two independent and nonparametric groups) and the plots creation were performed using the software R.

## 3 Results

### 3.1 Apparent specific growth rate derived from calorimetric measurements

Calorimetrically derived $\mu_{\mathrm{app}}$ were calculated as the slope of the curve ln(*P*(*t*)) vs. time during the exponential growth phase from *P_m_*. Details are given in the SM. Table 4 compares $\mu_{\mathrm{app}}$ determined with the different IMCs using closed ampoules after amendment with glucose.

**Table 4:** Apparent specific growth rate for different calorimeters.

IMC	*LOD_V_* mW L^−1^	$\mu_{\mathrm{app}}\;$ h^−1^	Standard error h^−1^
MC-Cal/100P	1	0.145^a^	0.007
TAM Air	0.2	0.138^ab^	0.004
TAM III	0.05	0.131^b^	0.001

The instruments with a medium *LOD_V_* (TAM Air) *μ*_app_ = (0.138 ± 0.008) h^−1^ and a high *LOD_V_* (MC-Cal/100P) *μ*_app_ = (0.144 ± 0.013) h^−1^ show statistically the same $\mu_{\mathrm{app}}$ , whereas the low *LOD_V_* instrument (TAM III) *μ*_app_ = (0.131 ± 0.003) h^−1^ provides a slightly smaller value. Although the difference between the results obtained with MC-Cal/100P and TAM III was small, it was significant.

### 3.2 Influence of sample size and calorimetric instrument on specific metabolic heat

**Figure 2:**
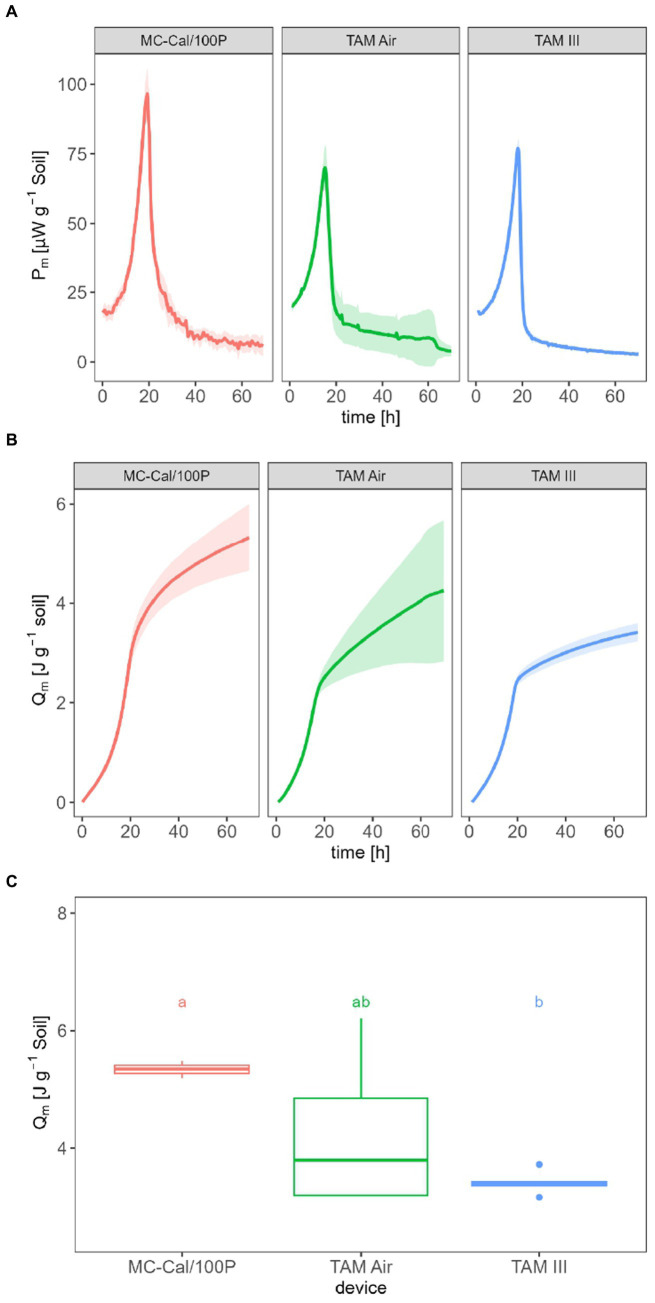
Specific heat production rate *P*_*m*,SG_ **(A)**, specific heat *Q_m_* **(B)** and *Q_m_* after 70 h of glucose-amended soil for the three applied calorimeters with different *LOD_V_* **(C)**.

*P* from soil amended with glucose solution were measured with three calorimeters differing in *LOD_V_*. *Q* resulted from the integration of *P* (Eq. 4). Figure 2A illustrates *P_m_*_,SG_ in μW g^−1^ soil. For MC-Cal/100P, a peak maximum of (98.0 ± 8.6) μW g^−1^ is observed after approx. 19.2 h. For TAM Air, *P_m_*_,SG_ reaches its peak maximum at (70.0 ± 7.7) μW g^−1^ after approx. 15.2 h. *P_m_*_,SG_, measured by TAM III reached (77.1 ± 3.0) μW g^−1^ after approx. 18.1 h.

Figure 2B displays the *Q_m_* in J g^−1^, with values of (5.34 ± 0.69) J g^−1^ for MC-Cal/100P, (4.25 ± 1.42) J g^−1^ for TAM Air, and (3.42 ± 0.18) J g^−1^ for TAM III. A statistically significant difference was found only between the IMC with a low *LOD_V_* (TAM III) and the IMC with a high *LOD_V_* (MC-Cal/100P), as seen in Figure 2C.

### 3.3 Influence of aeration on the thermal signal

**Figure 3:**
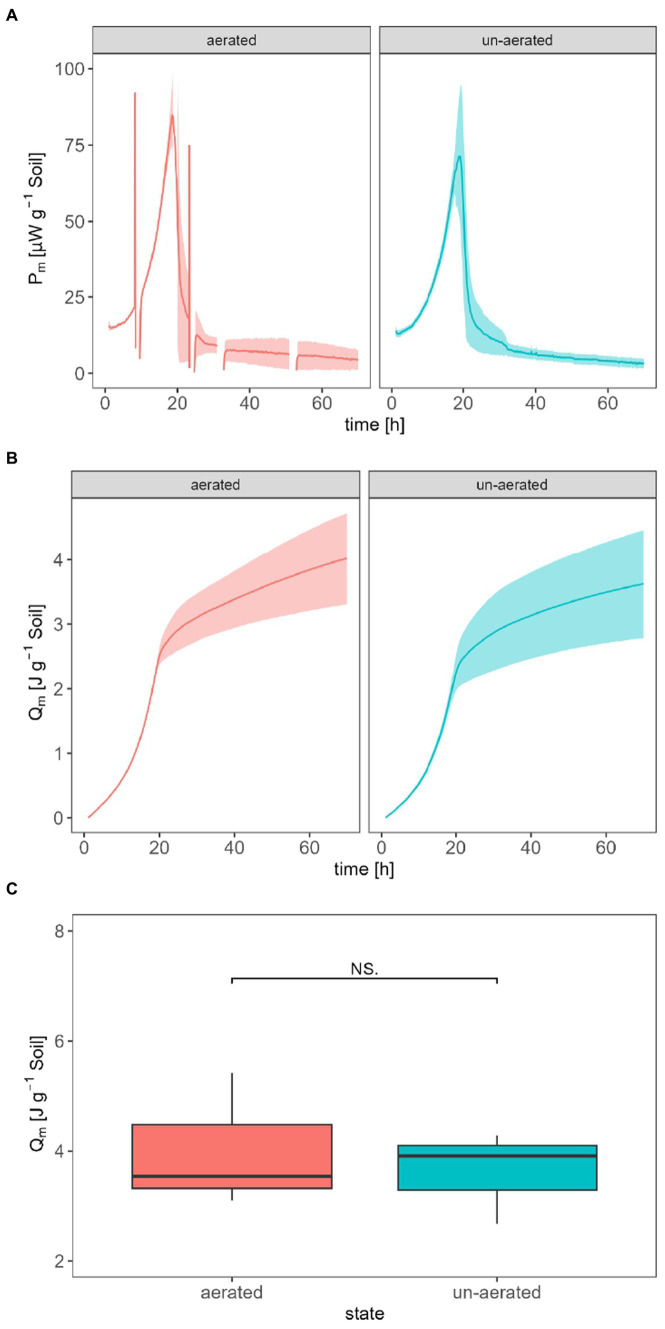
Influence of aeration on the specific heat production rate *P*_*m*,SG_ **(A)**, the specific heat *Q_m_* **(B)**, and the statistic comparison of *Q_m_* after 70 h for aerated and un-aerated treatment **(C)**.

The following comparison intends to answer the question of whether aerating calorimetric ampoules to prevent oxygen depletion affects the thermal signal. For better comparability, the experiments were performed with the IMC with the lowest *LOD_V_* (TAM III) adding glucose for two different treatments (aerated vs. un-aerated). The ampoules were aerated for 5 min, causing a thermal disturbance which lasted for approximately 2 h. To integrate the *P* (for obtaining the *Q*), the discontinuities caused by opening of the ampoule were mathematically treated by a linear interpolation of the signal during this time. However, if we focus on the interpolated signal, there was no statistically significant difference between the permanently un-aerated and transiently aerated treatments. The maximum *P_m_*_,SG_ for the un-aerated treatment was (71.4 ± 19.8) μW g^−1^ after 18.9 h, and (84.8 ± 11.7) μW g^−1^ after 18.7 h for the aerated treatment (Figure 3A). *Q_m_* for the un-aerated and aerated treatment was (3.62 ± 0.84) J g^−1^ and (4.02 ± 1.22) J g^−1^ after 70 h (Figure 3B), respectively. *Q_m_* does not show a significant difference (Figure 3C).

### 3.4 Influence of the aeration on the calorimetrically derived CO_2_ evolution rate

**Figure 4:**
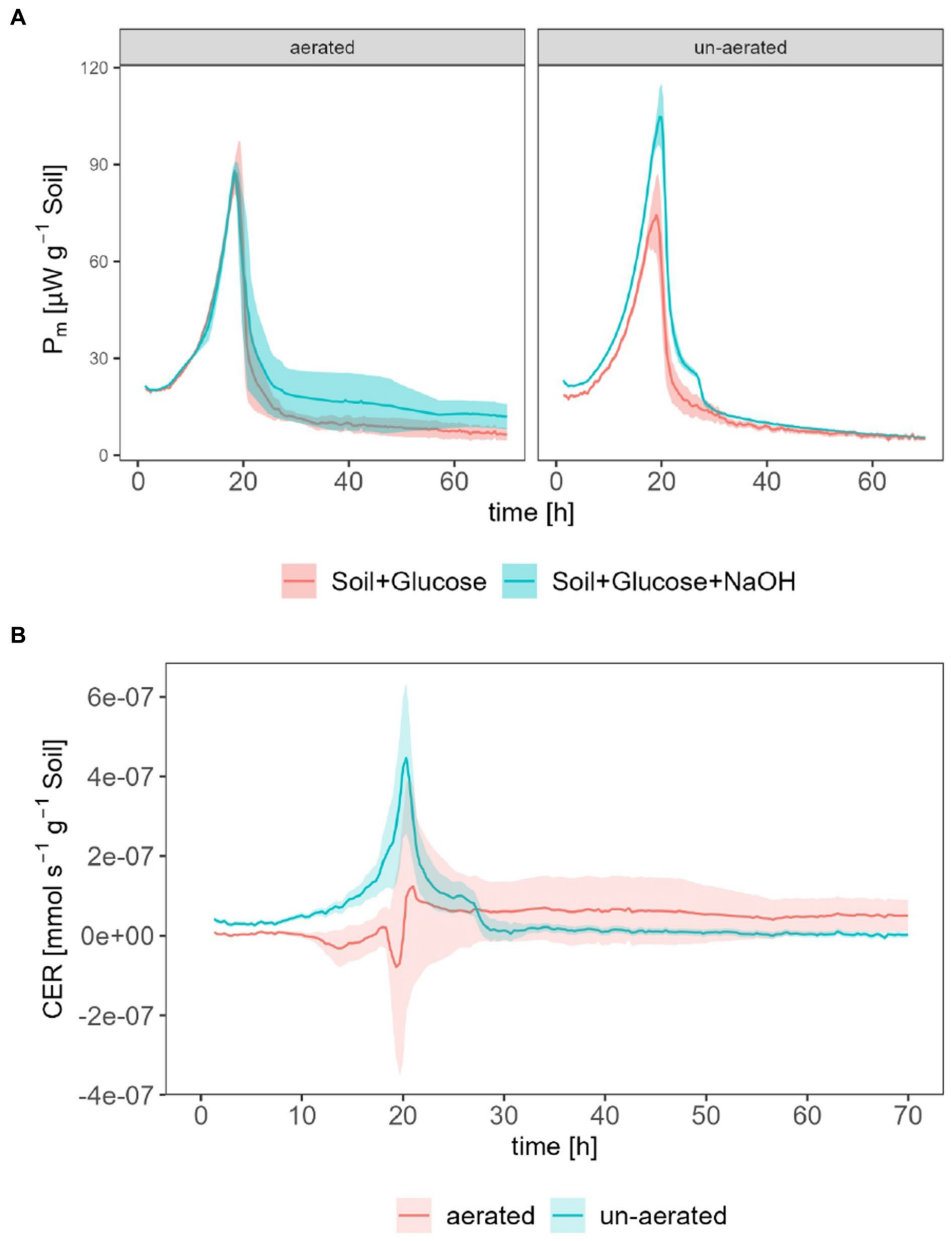
Calorimetrically derived CER; **(A)** shows *P*_m_ for measurements with (*P*_*m*,SGN_) and without (*P*_*m*,SG_) CO_2_ traps for aerated and un-aerated systems, **(B)** shows the derived CER for aerated and un-aerated systems.

The following comparison intends to reveal the impact of aeration of calorimetric ampoules on the calculation of calorimetrically derived CER. This comparison was done with the same instrument (TAM III) and glucose concentration as in the previous experiment. The opening of ampoules led to an increase in peak *P_m_*_,SG_ for soil amended with glucose (87.1 $\mu W\;g^{-1}$ at *t* = 18.7 h) compared with un-aerated ampoules (74.5 $\mu W\;g^{-1}$ at *t* = 19.0 h) but a decrease in peak *P_m_*_,SG_ for soil amended with glucose equipped with CO2 traps (88.0 $\mu W\;g^{-1}$ at *t* = 18.3 h) in comparison with un-aerated ampoules (105 $\mu W\;g^{-1}$ at *t* = 19.7 h) as shown in Figure 4A.

The CER was calculated according to Eq. 11. In the un-aerated ampoules, the CER reached a peak maximum of 4.47·10^−7^ mmol s^−1^ g^−1^ at *t* = 20.3 h. In the aerated case, the CER decreased below 0 since *t* = 10.7 h and increased until the peak value, which equals 1.24·10^−7^ mmol s^−1^ g^−1^ at *t* = 21 h, which was approx. 25% of CER in the un-aerated systems. After approx. 25 h, CER for un-aerated systems tended to around 0 mmol s^−1^ g^−1^ whereas CER for un-aerated system remained at approx. 5.00·10^−8^ mmol s^−1^ g^−1^.

### 3.5 Calorespirometric ratio of dynamic or integrated signals

**Figure 5:**
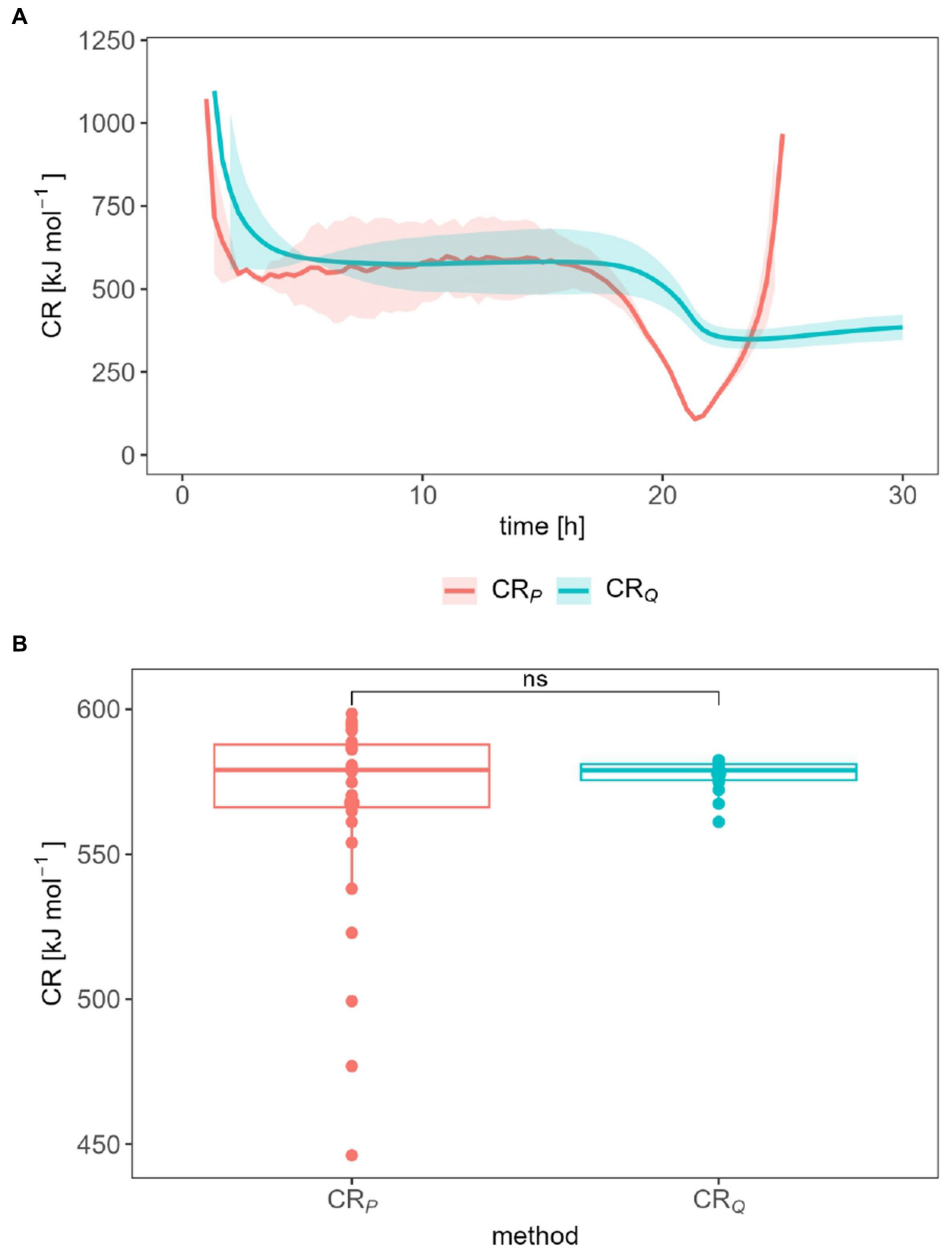
Influence of the data treatment on the CR **(A)** and statistical measurement error of the CR determination **(B)** during the exponential growth phase.

The CR was calculated either from *P* (Eq. 9) or *Q* (Eq. 10) observed during the exponential growth phase. The CR was 568 kJ mol^−1^ and 578 kJ mol^−1^ during exponential phase (8.48–19.0 h) for *P*-derived and *Q*-derived method, respectively (Figure 5A). Figure 5B shows that there is no statistically significant difference between the CR ratio calculated by both methods. The distribution of all CR data points also reflects the dispersion of this value around the average CR. Nevertheless, CR derived from the heat production rate started to decrease to 108.8 kJ mol^−1^ and then increased sharply again. CR derived from total heat presented a slight and smooth drop, it reached approx. 375 kJ mol^−1^ after 30 h. For CR, we focus on the first 30 h only, because thereafter, both the heat signal as well as the calorimetrically derived CER had dropped so much that only a very uncertain CR ratio resulted.

## 4 Discussion

Independent of the technical, preparative and data processing level of the calorimetric measurements, calorimetry delivers reliable and accurate key parameters such as *∆*_r_*H* for a better understanding of the relations between matter and energy fluxes in soil systems. Other essential key parameters such as CUE, CR, CER, and *μ*_app_, can reliably and practically be derived from calorimetric measurements for growth on rapidly metabolized substrates.

However, if over- and misinterpretations of calorimetric results are to be avoided in future thermodynamic soil research, the following questions need to be addressed. How accurately can the respective parameters be determined under the best measuring conditions? What influences do technical conditions, sample preparation and data evaluation exert on the results? What are the consequences of the calorimetric measurement accuracy for the derived parameters such as CUE, CR, CER, EUE, *μ*_app_, and $\eta_{A}$ ? This will be discussed in the following using the respective parameters.

### 4.1 Kinetic data interpretation

In principle, *P* corresponds to the product of a reaction rate and the associated reaction enthalpy (Eq. 3). $\Delta_{r}H\;$ is linked to the reaction stoichiometry via the law of Hess (Eq. 5). Calorimetric experiments thus provide both kinetic and stoichiometric information. Here, we first discuss the kinetic information expressed by $\mu_{\mathrm{app}}$ , calculated from the heat production rates during the exponential growth phase. Table 4 shows *μ_app_* with the corresponding error for the different IMCs with respective *LOD_V_*. All calorimetrically derived $\mu_{\mathrm{app}}$ values are similar and exhibit good agreement within the range reported in previous studies with comparable experimental setups (Barros et al., 2000; Koga et al., 2003). Comparison with literature values has its limits because the kinetics of soil processes depend on physical conditions, microbial communities, SOM, minerals etc. However, our values (Table 4) are in the upper range of the literature data from 0.035 h^−1^ to 0.157 h^−1^ (Barros et al., 2000). The *μ*_app_ values derived from the instruments with a low and medium *LOD_V_* (TAM III and TAM Air) cannot be statistically distinguished. However, the *μ*_app_ value obtained from MC-Cal/100P was significantly higher than the value derived from the TAM III (Table 4). Our observed variations in *μ*_app_ are assumed to be influenced by the interactive effects of the calorimeter’s thermal limits of detection and the sample sizes employed, which are expressed by the parameter *LOD_V_*. The literature data used for the comparison of *μ*_app_ were determined using a TAM 2277 with a thermal *LOD* of 0.15 mW and a calorimetric vessel of 5 mL and therefore a *LOD_V_* of 30 mW L^−1^. The difference in signal drift between TAM III (< 0.2 μW over 24 h) and MC-Cal/100P (< 40 μW over 24 h) could be a further reason for the observed deviation.

### 4.2 Determination of the metabolic heat

The second point to be discussed is the metabolic heat. In the case of *Q_m_*, the results obtained with the IMCs with different *LOD_V_* are within a small range, spanning from 3.42 to 5.34 J g^−1^ (Figure 2). By considering the amendment of 900 μg (0.005 mmol) of glucose per g-DW soil or 0.0043 mmol per g-wet soil and the combustion enthalpy of glucose (−2,808 kJ mol^−1^) (Kabo et al., 2013), a maximum enthalpy input into the soil of 12.1 J g^−1^ can be calculated. Assuming that (i) the difference between these energy values represents the energy content of freshly formed biomass, (ii) all added glucose is completely consumed, and (iii) energy contributions from SOM or necromass can be neglected, an energy use efficiency (EUE) between 55.9 and 71.7% is obtained using Eq. 6. These values are at the upper end of reported data in soil, which range from 15.6 to 63.1% (Barros and Feijoo, 2003).

Significant differences in *Q_m_* were observed between MC-Cal/100P and TAM III, as depicted in Figure 2C. Once again, the different *LOD_V_* values can be considered as potential reasons for these discrepancies. Therefore, it is recommended to use the instrument with the lowest *LOD_V_* provided that the size of the soil sample is large enough to obtain homogeneous replicates.

**Figure 6:**
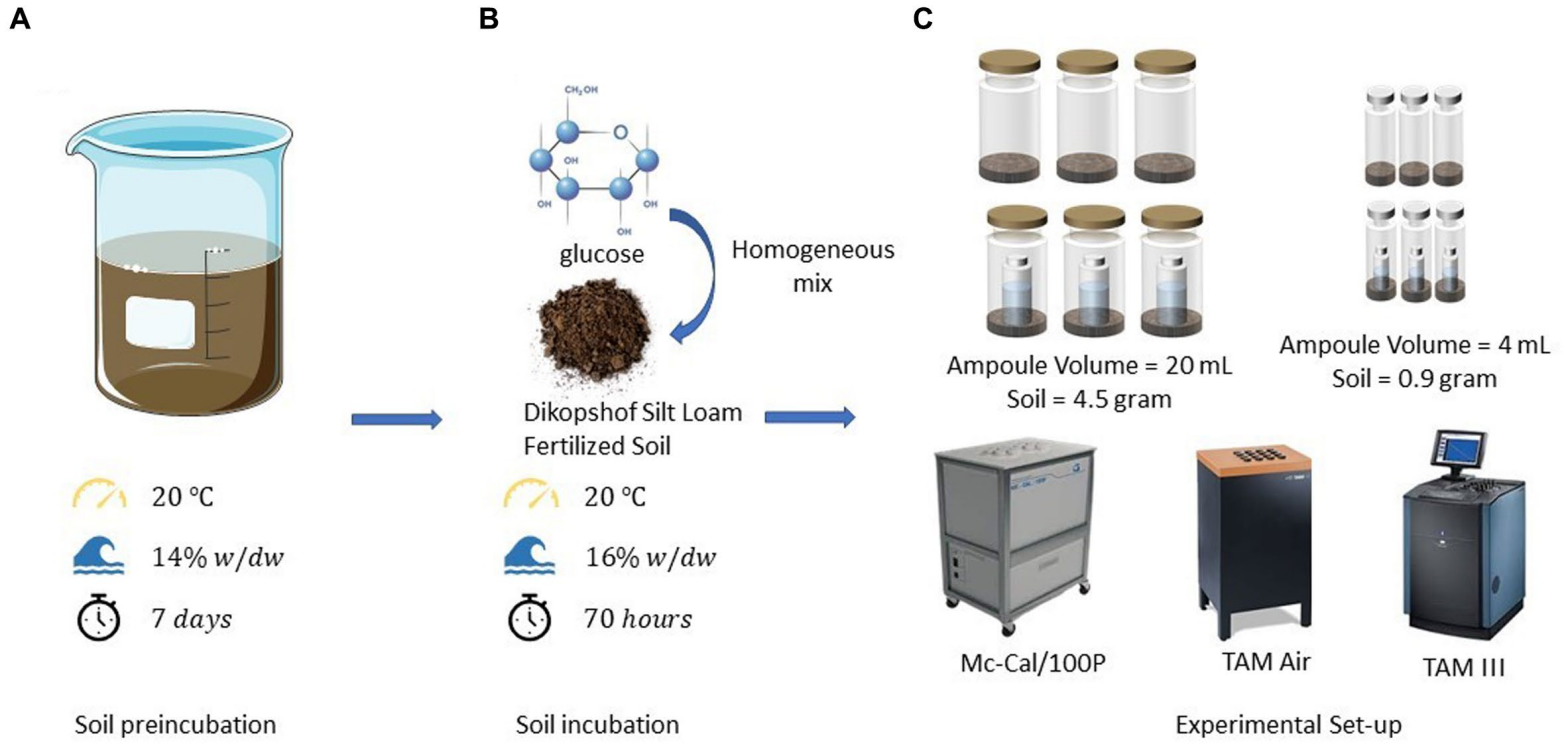
**(A)** Pre-incubation conditions and duration of the experiments, **(B)** environmental conditions, procedures, materials, and durations for soil incubations, and **(C)** experimental set-ups and devices utilized in calorimetric incubation experiments.

Aeration of the calorimetric ampoules may be necessary to replenish the consumed oxygen and remove the evolved CO_2_ to prevent adverse effects on microbial activities (Figure 6). It has already been reported that oxygen depletion and the accumulation of CO_2_ in the headspace inhibit microbial activity (Neilson and Pepper, 1990). Figure 3A demonstrates that aeration introduces some thermal disturbances causing discontinuities in the thermal signal; however, no statistically significant difference was observed between aerated and non-aerated measurements (Figure 3C). This is surprising as about three-fourth (75%) of the oxygen in the ampoules might have been used assuming complete mineralization of the added glucose (see Table 2). Such a strong reduction of the oxygen concentration should have resulted in a decrease in aerobic microbial activity and a shift toward anaerobic processes, which should have been reflected in the heat signal. Obviously, a sufficiently large soil volume remained aerobic to support the observed unchanged heat production. During the integration of the heat production rate, the disturbances were mathematically treated by linear interpolations between the undisturbed signals, making data evaluation more complex. Hence, whenever possible, it is advisable to avoid opening the ampoules. To make decisions regarding whether an ampoule should be aerated or not, the calculations discussed in section 2.1 can be consulted.

### 4.3 Determination of the carbon dioxide evolution rate in the calorimeter

The shape of the calorimetrically derived CER, as depicted in Figure 4, supports the concept of utilizing the difference in heat production rates with (*P*_SGN_) and without (*P*_SG_) CO_2_ traps in closed systems. However, during the exponential growth phase, inconsistencies were observed in the calorimetrically derived CO_2_ values for regularly opened ampoules, particularly with unexpectedly negative values of CER. While the exact reasons for these observations are unknown, several factors may have contributed. Firstly, the aeration of channels led to temperature fluctuations in response to the ambient environment, resulting in arbitrary and unpredictable heat production rate measurements (up to 0.45 W) within a short time (approx. 2 h). This necessitated omission of data and interpolation, introducing the potential for manual and non-reproducible errors during the data analysis process. Additionally, the calorimeter required a certain amount of time to return to its original signal level following the opening of calorimetric ampoules and returning to measuring channels. This delay in returning to the original signal level could potentially introduce deviations in the measured data, particularly for short-term experiments. Furthermore, previous studies confirmed this deviation in CER by inserting and removing CO_2_ traps at a regular time interval (Barros et al., 2011). These findings align with the observations made in this study regarding inconsistencies in the calorimetrically determined CER.

Another significant factor to consider is the act of opening the ampoules, which exchanges the air inside the ampoule with ambient air (as shown in Figure 6). These two atmospheres both differ in temperature and water content. When the ampoule is closed again, this can lead to the evaporation of water, resulting in an associated endothermic effect. The highest endothermic impact of opening the ampoule (15.3 μW) was observed in experiments involving glucose and the CO_2_ trap. Using the evaporation enthalpy of water at 20°C (44.2 kJ mol^−1^, Hodgman, 1951), we can estimate an evaporation rate of 0.35·10^−9^ mol s^−1^ or 6.23·10^−9^ g s^−1^. The water content of the air space in the applied ampoule after water saturation, assuming equilibrium, is 17.3 g m^−3^ at 20°C (Hodgman, 1951), corresponding to 6.19·10^−5^ g in the vial. Taking this value and dividing it by the evaporation rate results in an evaporation duration of 2.76 h. Consequently, the observed maximum endothermic deviation could explain a vaporization of 2.76 h until the saturation of completely dry air is achieved. Although this rough estimate does not take into account the substantial water content in the soil sample (0.144 g), as its vaporization extent is more difficult to estimate, it emphasizes the importance of water evaporation on the heat signal.

Lastly, the absorption of CO_2_ by NaOH leads to a reduced partial pressure of CO_2_. This reduction has the potential to interfere with the growth of specific microorganisms that rely on CO_2_ fixation as a vital component of their growth. However, due to the regular aeration, the system becomes dynamic, preventing CO_2_ accumulation and avoiding limitations in O_2_ availability for growth. These factors may contribute to varying outcomes in C mineralization, as observed in a study by Hopkins (2008). On the other hand, several studies have explored the comparison of respiration rates in soil systems between well-aerated and static closed systems. Their findings demonstrated that well-aerated systems yielded higher respiration rates than static closed systems (Sakamoto and Yoshida, 1988; Jensen et al., 1996; Rochette et al., 1997; Suh et al., 2006).

It is important to consider these factors when interpreting and analyzing the CO_2_ data in closed, static calorimetric experiments, as they can introduce uncertainties and potential sources of error. To sum up, it is not advisable to aerate the ampoules during experiments when oxygen is not a limiting factor for soil microorganisms. Opening the ampoules can introduce biases in the calorimetrically derived CO_2_ results, affecting the accuracy and reliability of the measurements. The question of whether oxygen could potentially be limiting can be estimated by calculating the oxygen content of the ampoule.

To achieve simultaneous measurement of *P* and CO_2_ with minimal disturbance, it is necessary to explore alternative approaches. The combination of the calorimetric measurement principle with a Warburg apparatus might be a solution. The conventional Warburg apparatus is a device for measuring the pressure of a gas at constant volume and constant temperature so that the pressure is a measure of the quantity of gas and changes in pressure reflect the production and absorption of gas (Oesper, 1964). Another option might be the incorporation of a CO_2_ sensor into the calorimetric ampoule, if the potential heat evolution of the sensor itself does not interfere with the measuring signal. Wadso (2015) developed a new calorimetric-respirometric ampoule using a valve on the ampoule that allows opening and closing (aeration) inside the calorimeter for short-term processes. As a result, the calorimetric measurement is not disturbed and gives more reliable results. Calorimetric ampoules need to be covered to prevent water evaporation interfering with the calorimetric signal by the large evaporation enthalpy of water. However, the calorimetric ampoule could be closed with gas separation membranes being impermeable to water but allowing the transport of oxygen (Valappil et al., 2021). Both ideas could be part of future calorespirometer developments.

### 4.4 Influence of data evaluation on the calorespirometric ratio

The CR shows a similar range between 100 and 1,200 kJ mol^−1^ and trends regardless of the evaluation method used. CR between 0 and 600 kJ mol^−1^ and extreme values of 1,500 kJ mol^−1^ are reported (Hansen et al., 2004). The CR value drops in the first 3 h and is then approximately constant until the 18th or 20th hour. When the heat production rate decreases, so does the CR. After the 20th to 21st hour, the CR (derived from the heat production rate) increases or remains constant at about 375 kJ mol^−1^. Thus, our trend is similar to those observed by Barros et al. (2010). The CR drop at the beginning of the measurement should be considered carefully. Currently, both signals (*P_m_* and CER or *Q_m_* and accumulated CO_2_) are very small and thus the quotient of the two quantities is strongly error prone. The same applies to the signals after the 20th hour. The constant average CR of 577.7 kJ mol^−1^ (from *Q_m_*) or 567.6 kJ mol ^−1^ (from *P_m_*) speaks for a constant growth stoichiometry. For these values, the signal evaluation seems to be without relevance.

### 4.5 Limitations of the informative content of the calorespirometric ratio

Assuming an aerobic metabolism and the validity of the oxycaloric equivalent (−455 kJ mol^−1^ O_2_) (Gnaiger and Kemp, 1990), the CUE can be calculated from the measured CR (Hansen et al., 2004; Colombi et al., 2022). For that purpose, we extended the equation of Hansen et al. (2004) by including the enthalpy of the nitrogen source (NH_4_^+^, Eq. 13) to complete the energy balance. The derivation of Eq. 13. is provided in the supporting material.


(13) $$CUE=\frac{\frac{4\cdot CR}{455\;\mathrm{kJ}\;{\mathrm{mol}}^{-1}}-\gamma_{S}}{n_{N}^{X}\cdot \gamma_{N}-\gamma_{X}+\frac{4\cdot CR}{455\;\mathrm{kJ}\;{\mathrm{mol}}^{-1}}}\;$$

Here $\gamma_{S}$ , $\gamma_{N}$ , $\gamma_{X}$ stand for the relative degree of reduction of the substrate, the nitrogen source, and the biomass, respectively. $n_{N}^{X}$ stands for the molecular nitrogen content of biomass. If we now ask ourselves what measurement accuracy is required for CR in the context of this theory in order to achieve a certain accuracy for CUE, we need to look at the derivative of CUE with respect to CR (Eq. 14).


(14) $$\begin{array}{l} \Delta CR=\left|\frac{dCR}{dCUE}\right|\Delta CUE=\\ \;\;\;\;\;\;\;\left|\frac{n_{N}^{X}\cdot \gamma_{N}-\gamma_{X}+\gamma_{S}}{4\cdot {\left(1-CUE\right)}^{2}}\cdot \;455\;\mathrm{kJ}\;{\mathrm{mol}}^{-1}\right|\Delta CUE\; \end{array}$$

**Figure 7:**
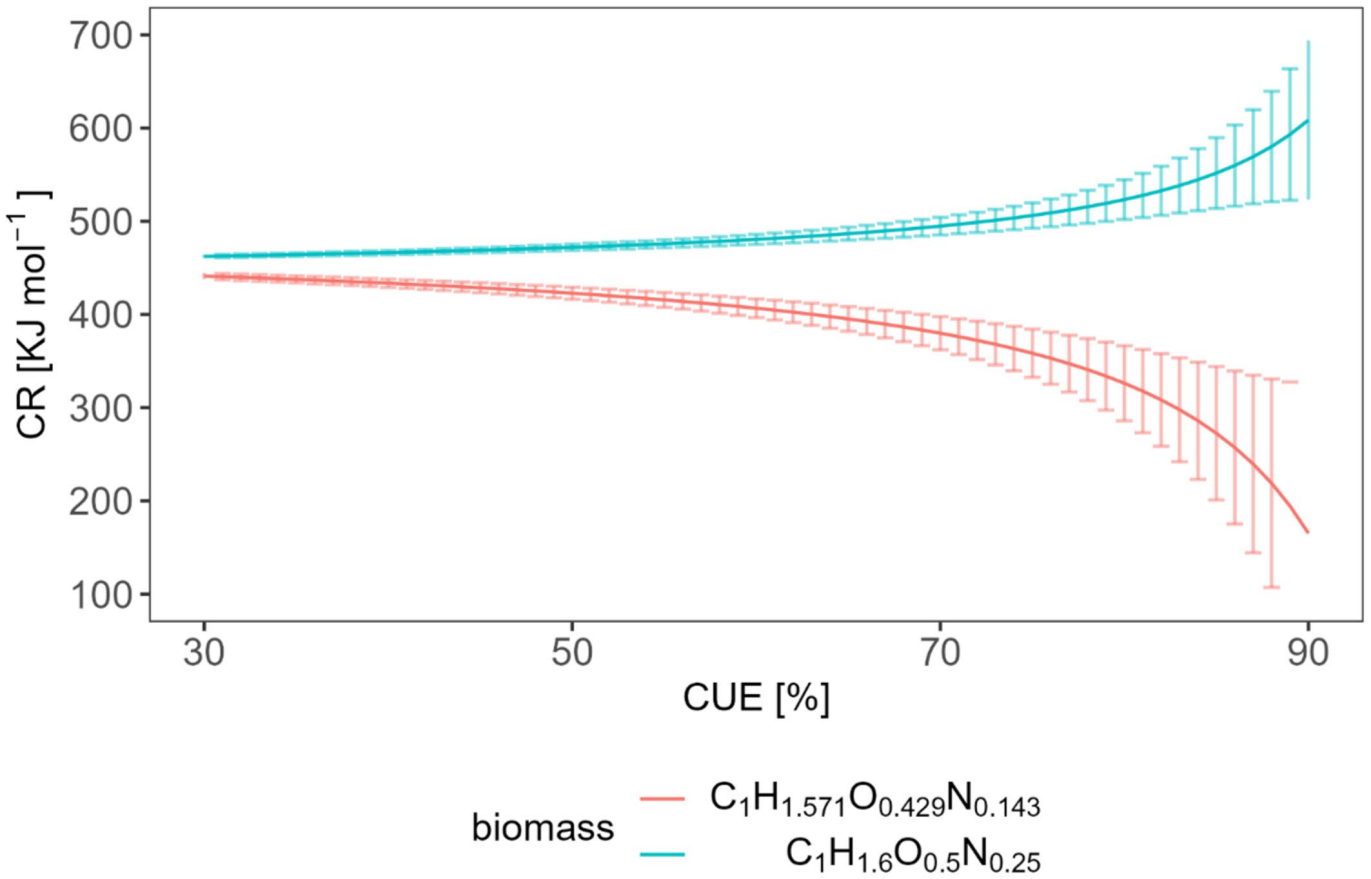
Expected relation and uncertainty between the CR and the CUE with two different biomass compositions.

Figure 7 depicts this relation and the uncertainty (assuming that the CUE needs to be determined with 5% accuracy) for two different biomass compositions. We considered two elemental biomass compositions because the C/N in soil microbial biomass is different from that growing in liquid culture. A C:N ratio of 7:1 is a generally accepted average for soil (Xu et al., 2013; Mooshammer et al., 2014). Different biomass compositions cause different $\gamma_{X}$ and thus different combustion enthalpies of the biomass (see SM). C_1_H_1.6_O_0.5_N_0.25_ is suggested for bacteria growing in liquid culture in bioreactors (Babel et al., 1993), while C_1_H_1.571_O_0.429_N_0.143_ takes the C/N ratio in soil microbial biomass into account.

The average CR we obtained from heat measurements was 577.7 kJ mol^−1^, and CR from heat production rate measurements was 567.6 kJ mol^−1^. These values correspond to CUE values of 0.878 or 0.868, respectively, which seem unrealistically high. These calculations were based on a biomass composition of C_1_H_1.6_O_0.5_N_0.25_. However, when considering a biomass composition of C_1_H_1.571_O_0.429_N_0.143_, such high CR values become simply impossible. Qiao et al. (2019) reports about a wide range of CUE with a global average of 0.5 ± 0.25. However, the CUE obtained with glucose also depended on the applied method. For instance, the CUE tended to be lower (<0.4) under identical incubation conditions using ^18^O incorporation and stoichiometric modeling. Substrate-dependent ^13^C-based methods, calorespirometry, and metabolic flux modeling provides often higher CUE (>0.6) (Geyer et al., 2019). Barros et al. (2010) reported CUE values in the range of 0.75–0.77 applying the same method as in our study. Neglecting the simultaneous metabolism of SOM components could be a potential reason for the high CUE values derived from Eq. 13.

Furthermore, Eq. 13 assumes that oxygen is the terminal electron acceptor. Therefore, it is only applicable in oxic, non-water-saturated soils, where glucose and other sugars derived from starch or (hemi-)celluloses are mainly oxidized with oxygen as the terminal electron acceptor.

**Figure 8:**
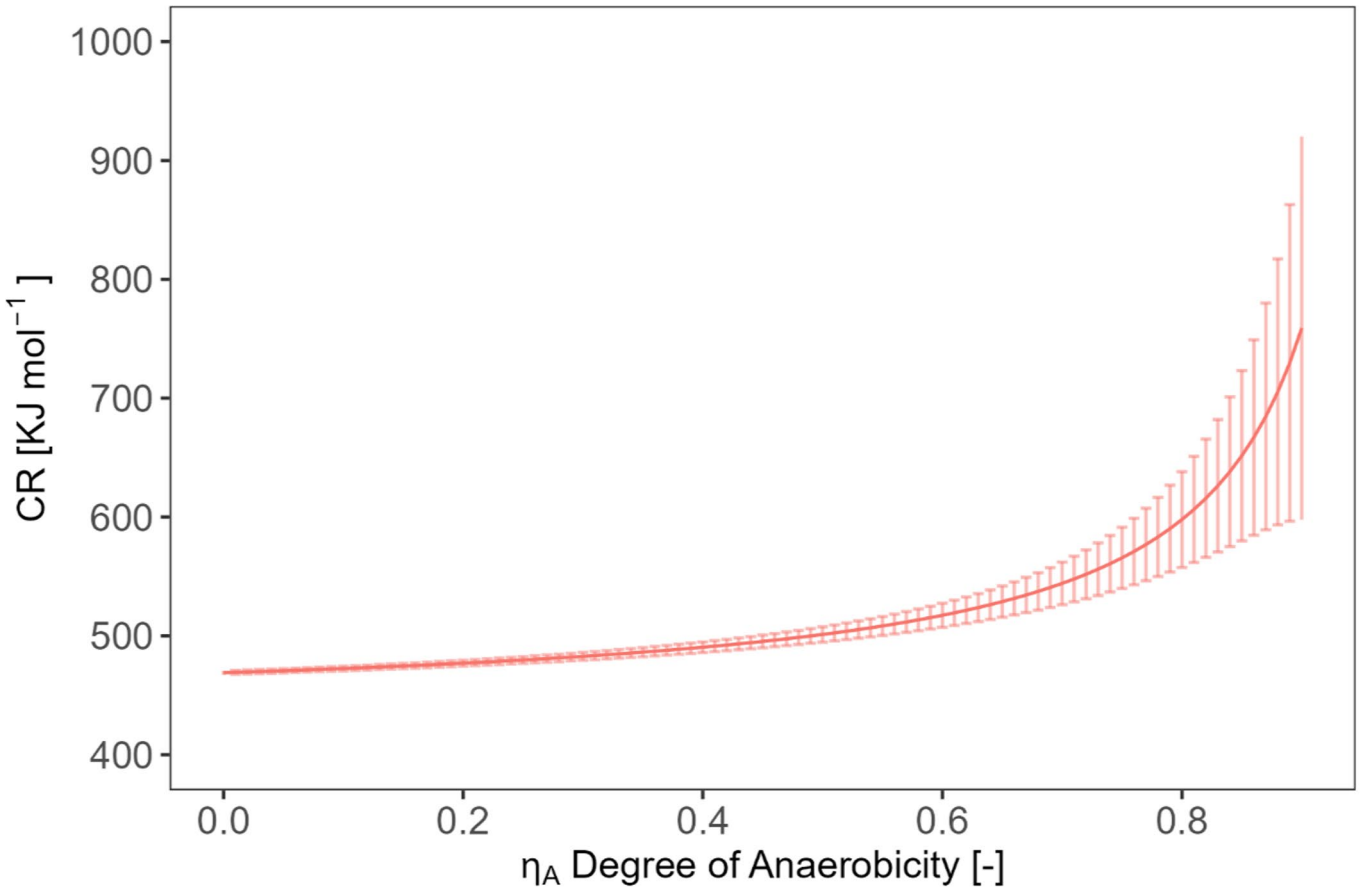
Expected relation and uncertainty between the CR and the degree of anaerobicity.

In soils under partially anoxic conditions, the theory cannot be applied. Soil redox conditions can strongly fluctuate both temporarily and spatially. For instance, after (heavy) rain events, the topsoil becomes partly water-saturated for a period, leading to a quick limitation of oxygen. Furthermore, anoxic conditions can exist even at microsites in soil due to the combination of high microbial activities and slow oxygen diffusion. The influence of anaerobic metabolism on CR will be discussed below using a combination of the acetate fermentation (C_6_H_12_O_6_ + 2H_2_O → 2C_2_H_4_O_2_ + 2CO_2_ + 4H_2_) with the acetogenesis (2CO_2_ + 4H_2_ → C_2_H_4_O_2_ + 2H_2_O) yielding the reaction shown in Eq. 15. Acetate is a good example because it is often formed in soil under anoxic conditions. In order to analyze the influence of the transition from oxic to anoxic conditions on the CR, we consider the combination of the catabolic oxic glucose oxidation and anoxic conversion to acetate expressed as the degree of anaerobiosis, $\eta_{A}$ , ranging from 0 (complete oxic conditions) to 1 (complete anoxic conditions) (Figure 8, Eq. 15). The $\Delta_{\mathrm{OX}}H$ represents the combustion enthalpy of glucose (−2813.6 kJ mol^−1^) and $\Delta_{\mathrm{ANOX}}H$ were calculated using the law of Hess [−((3·-873.4) +2813.6) = −193.4 kJ mol^−1^] and the combustion enthalpy of glucose and of acetate (−873.4 kJ mol^−1^). The combustion enthalpies of compounds in the water dissolved state was taken from (von Stockar et al., 1993).


$$\begin{array}{ccc} \mathrm{Oxic metabolism} & \begin{array}{l} C_{6}H_{12}O_{6}+6\;O_{2}\to 6{\mathrm{CO}}_{2}+6\;H_{2}O\\ \Delta_{\mathrm{OX}}H=-2813.6\;\mathrm{kJ}/\mathrm{mol} \end{array} & \\ \mathrm{Anoxic metabolism} & \begin{array}{l} C_{6}H_{12}O_{6}\to 3{\mathrm{CH}}_{3}\mathrm{COOH}\\ \Delta_{\mathrm{ANOX}}H=-193.4\;\mathrm{kJ}/\mathrm{mol} \end{array} & \end{array}$$

(15) $$CR=\frac{\eta_{A}\cdot \Delta_{\mathrm{ANOX}}H+\left(1-\eta_{A}\right)\cdot \Delta_{\mathrm{OX}}H}{\left(1-\eta_{A}\right)\cdot 6}$$

The maximum error of CR caused by the error of $\eta_{A}$ is estimated using Eq. 16.


(16) $$\Delta CR=\left|\frac{dCR}{d\eta_{A}}\right|\Delta \eta_{A}=\left|\frac{\Delta_{ANOX}H}{6{\left(1-\eta_{A}\right)}^{2}}\right|\Delta \eta_{A}$$

Our simple model (Eqs. 14, 16) allows estimating the required accuracy in measuring CR to obtain statements with an error < 5% on CUE or $\eta_{A}$ . For example, if we aim to determine a typical CUE of 0.5 with 5% error for a biomass composition C_1_H_1.6_O_0.5_N_0.25_, we will need to measure a CR value with an accuracy of (472.1 ± 3.4) kJ mol^−1^. In the case of a biomass composition of C_1_H_1.571_O_0.429_N_0.143_, a CR value of (422.8 ± 6.4) kJ mol^−1^ would be required. Similarly, if our goal is to calculate $\eta_{A}$ with a value of 0.5 and 5% error using CR, we should be able to measure CR values with an accuracy of (501.2 ± 10.1) kJ mol^−1^ (assuming a biomass composition of C_1_H_1.6_O_0.5_N_0.25_). However, the actual measuring error is 5.52 kJ mol^−1^ using the integrated values or 21.6 kJ mol^−1^ using the rates (interquartile range). The measuring error obtained with integrated value falls within the range of requirements while using the rates not fit the requirement. It is important to note that the substrate we analyzed in our test had a high conversion rate, which may have contributed to these relatively favorable evaluations. More complex substrates with lower energy content or slower conversion rates will lead to smaller signals which are more influenced by the signal noise and more difficult to integrate. Such material calls for higher accuracy in measuring CR to achieve the desired precision in estimation CUE and $\eta_{A}$ .

In conclusion, the simple model offers valuable insights into the required measurement accuracy for determining CUE or $\eta_{A}$ with a given error margin. However, the complexity of real-world scenarios, including variations in biomass composition and substrate characteristics, demands careful consideration and further investigation to ensure accurate and reliable estimations as well as striving for the development of improved calorespirometers.

## 5 Conclusion

The calorimetric determination of the apparent specific growth rate $\mu_{\mathrm{app}}$ , the metabolic heat *Q* simultaneously with the CER via CO_2_ trap method is possible and provides plausible data for an easily degradable substrate (e.g., glucose) added to the soil. However, several variables affect the results. Firstly, volume-related declared thermal limit of detection (*LOD_V_*) represents the integrated effect of thermal calorimeter sensitivity and sample size. In order to obtain more reliable and reproducible data, it is recommended to use an IMC with a low *LOD_V_*. For calorimeters with a comparable *LOD_V_*, the instrument with a larger calorimetric ampoule should be preferred to better cover soil heterogeneities and to achieve results representative for the soil under study.

Regular aeration of the calorimetric vessel is considered as a method to counteract oxygen depletion. Despite the thermal disturbances caused by this, no significant differences in the thermal signal were observed between analyses with and without regular aeration. However, in the case of the simultaneous measurement of *P* and CER, the difference between the two calorimetric signals with and without CO_2_ traps must be evaluated. In such cases, the aeration of the calorimeter causes a significant error.

Equation 13 shows a tight link between the CR and the CUE. The comparison between accuracy requirements from this model for CR with the real errors of determination reveals that only in the best case the currently available instruments are sensitive enough to infer the CUE from the CR. The same holds true for the link between the CR and the proportion of anaerobic processes from the CR. With more complex substances such as polymeric carbohydrates, plant debris, non-viable bacteria, chitin etc., slower mass conversions and thus more error-prone CR values are to be expected. This means that novel types of calorimeters should be developed that either have better thermal sensitivity or allow larger soil samples to be measured. The second point is significant because a larger soil sample size can counteract the influence of soil heterogeneity on the thermal signal and is probably technically the most feasible. Furthermore, larger calorimetric ampoules facilitate the insertion of gas sensors for a better combination of calorimetry and respirometry. The analysis of small samples, however, might be interesting for the study of processes in selected microhabitats, e.g., the rhizosphere or different aggregate fractions.

The discrepancies between the CUE values or $\eta_{A}$ derived from the experimental CR values and the expectations from the models of Hansen et al. (2004) and Chakrawal et al. (2020) result from the simplifying basic assumptions of both models, which are discussed in section 4.5. This argues for the application of more complex numerical models, which include a certain proportion of anaerobic reactions, the usage of energy and “building blocks” from the SOM, the interaction of the OM with soil minerals, etc. The extension of the models to other factors will provide a better understanding of the intricate processes influencing carbon and energy utilization in soil systems.

The surprising result that a small variation of the elemental biomass composition can even change the trend of the CUE/CR relation shows that changes in the microbial community may not only affect the kinetics of the matter and energy fluxes as expected but also the process stoichiometry and thus the CUE/CR relation. Consequently, future numerical models should also take this effect into account.

## List of symbols

**Table:** 

Symbol	Property	Unit
CER	${\mathrm{CO}}_{2}\;$ evolution rate	mol g^−1^ s^−1^
CR	Calorespirometric ratio	J mol^−1^
CUE	Carbon use efficiency	mol mol^−1^
DW	Dry weight	g
EUE	Energy use efficiency	J J^−1^
*LOD_V_*	Volume-related thermal limit of detection	W L^−1^
*P*	Heat production rate	W
*P_m_*	Specific metabolic heat production rate	W g^−1^
*Q*	Heat	J
*Q_m_*	Specific total metabolic heat	J g^−1^
(S)OM	(Soil) Organic matter	g
*WHC*	Water holding capacity	g g^−1^
$\Delta_{\mathrm{abs}}H$	Enthalpy of absorption	J mol^−1^
$\Delta_{c}H$	Combustion enthalpy	J mol^−1^
$\Delta_{r}H$	Reaction enthalpy	J mol^−1^
$\eta_{A}$	Degree of anaerobicity	---
*μ*_app_	Apparent specific growth rate	s^−1^
**Subscripts**	**Meaning**	
a	aerated	
G	Glucose added	
N	Equipped with CO_2_ trap (NaOH)	
S	Soil	
u	un-aerated	

## Data availability statement

The link with the datasets is the following: https://doi.org/10.48758/ufz.14030.

## Author contributions

SY: Conceptualization, Data curation, Formal analysis, Investigation, Methodology, Validation, Visualization, Writing – original draft. ED: Conceptualization, Data curation, Formal analysis, Investigation, Methodology, Validation, Visualization, Writing – original draft. AR: Data curation, Writing – review & editing. HH: Supervision, Writing – review & editing. CF: Supervision, Writing – review & editing, Visualization. AM: Supervision, Writing – review & editing. MK: Supervision, Writing – review & editing. TM: Conceptualization, Data curation, Funding acquisition, Project administration, Supervision, Writing – review & editing.
