# Supplementary material for: Enhancing insights: exploring the information content of calorespirometric ratio in dynamic soil microbial growth processes through calorimetry
Source: Front Microbiol. 2024 Feb 2;15:1321059. doi: 10.3389/fmicb.2024.1321059 (PMC10869564; doi:10.3389/fmicb.2024.1321059)
Supplement: Supplementary file 1 [file Data_Sheet_1.pdf]

Supplementary Material

Enhancing Insights: Exploring the Information Content of Calorespirometric Ratio in Dynamic Soil Microbial Growth Processes through Calorimetry

Shiyue Yang[1], Eliana Di Lodovico[1,2], Alina Rupp[1], Hauke Harms[1], Christian Fricke[2], Anja Miltner[1], Matthias Kästner[1], Thomas Maskow[1*]

[1] Department of Environmental Microbiology, Helmholtz-Centre for Environmental Research – UFZ, Leipzig, Germany

[2] Rheinland-Pfälzische Technische Universität Kaiserslautern-Landau (RPTU) - Campus Landau, Fortstraße 7, 76829 Landau in der Pfalz, Germany

*** Correspondence:**
Thomas Maskow
thomas.maskow@ufz.de

1 Supplementary Calculations

1.1 Oxygen Limitation

The estimation of the oxygen limitation occurs assuming an ideal gas behavior.

$$\boldsymbol{n_{O2}} = \frac{\boldsymbol{\Pi \cdot V}}{\boldsymbol{\xi_{O2,air} \cdot R \cdot T}} \tag{1}$$

Here stands *Π*, *V*, $\xi_{O2,air}$, *R*, *T* for the presure (101 325 Pa), the gas volume of the ampoule, the mole fraction of oxygen in air (20.94%), the universal gas constant (8.314 J mol^{-1} K^{-1}) and the temperature in K. The maximum required oxygen can be estimated assuming a complete oxidation of glucose under fully anerobic condition.

The water content in our experiments was 16% (w/dw), so the amount of dry soil is

$$W_{dw} = \frac{4.5\ g}{1 + 0.16} = 3.88\ g$$

The density of dry soil particles is estimated at 2.65 g cm^{-3}. Therefore, the volume occupied by dry soil is:

$$V_{dw} = \frac{3.88\ g}{2.65\ \mathrm{g}\ {cm^3}^{-1}} = 1.46\ cm^3$$

The volume occupied by water equals to

$$V_{water} = \frac{3.88 \times 16\%}{1.00\ \text{g}\ cm^{3\ -1}} = 0.62\ cm^3$$

The volume of air space equals to

$$V_{air} = 20 - 1.46 - 0.62 = 17.92\ cm^3$$

Considering O_2 occupies 20.94% of air space, so the volume of oxygen in the head space equals to

$$V_{O_2} = 17.9192\ \times 20.94\% = 3.75\ cm^3$$

Here, we get for the available oxygen:

$$\text{n}_{O_2} = \frac{P \cdot V}{R \cdot T} = \frac{101325(Pa) * 3.7523 * 10^{-3}(L)}{8314\ \frac{Pa*L}{mol*K} * (273.15+20)K} = 1.56 \times 10^{-4}\ mol$$

Beside of the available oxygen, the required oxygen is required for the comparison. It was estimated assuming the worst case (i.e. complete combustion of the added glucose.

$$C_6H_{12}O_6 + 6O_2 \rightarrow 6CO_2 + 6H_2O$$

We added 900 µg glucose/g-DW soil. Therefore, the amount of glucose we added is

$$n_{glucose} = \frac{3.88\ g\ \times 0.9mg\ g^{-1}dw}{180.16\ mg\ mmol^{-1}} = 0.01938\ mmol$$

The maximum oxygen amount we need is 6*0.01938 = 0.11628 mmol <0.1559 mmol. Therefore, the oxygen is enough for aerobic combustion.

1.2 Maximum carbon dioxide concentration in %

Assuming the complete combustion of glucose, 0.116 mmol CO_2 is maximum produced:

$$n_{CO_2} = 6 \times 0.01938\ mmol = 0.1163\ mmol.$$

The same amount of oxygen is consumed, thus, the pressure in the ampoule does not change. The CO_2 volume is 2.685 mL.

$$v_{CO_2} = \frac{n \cdot R \cdot T}{P} = \frac{1.1163 \cdot 10^{-4}\ mol \cdot 8.31441\ J\ mol^{-1}\ K^{-1} \cdot 293.15\ K}{101325\ J\ m^{-3}} = 2.685\ m^{-3}$$
$$= 2.685\ mL$$

The maximum concentration of CO_2 of 15 % is therefore obtained by calculating the proportion of CO_2 volume in the total gas volume of the ampoule.

$$C_{CO_2} = \frac{2.685\ mL}{17.91\ mL} \cdot 100\% = 15\ \%$$

The known CO_2 content of the air of 400 ppm or 0.04% does little to change this estimate. The same results are valid for 4 mL volume ampoules for TAM III, because they are filled with the same ratio of soil/air..

1.3 Relationship between CR and CUE

1.3.1 Biomass-1: $CH_{1.6}O_{0.5}N_{0.25}$

If we relate glucose to 1 C-mole, the equation for glucose is CH_2O and for the growth reaction is as follows.

$$CH_2O + (Y_{O/S}) \cdot O_2 + (\mathrm{CUE} * X_N^X) \cdot NH_3 \rightarrow (1 - CUE) \cdot CO_2 + (CUE) \cdot CH_{1.6}O_{0.5}N_{0.25} + (2 + 3 * (\mathrm{CUE} * X_N^X) - (1.6 * CUE))H_2O$$

Taking the oxocaloric equivalent, the reaction enthalpy depends on the respective yield coefficient $Y_{O/S}$.

$$\Delta H_r = -455\ \frac{kJ}{mol} \cdot Y_{O/S}$$

1.3.2 Biomass-2: $CH_{1.571}O_{0.429}N_{0.143}$

$$CH_2O + (Y_{O/S}) \cdot O_2 + (\mathrm{CUE} * X_N^X) \cdot NH_3 \rightarrow (1 - CUE) \cdot CO_2 + (CUE) \cdot CH_{1.571}O_{0.429}N_{0.143} + (2 + 3 * (\mathrm{CUE} * X_N^X) - (1.6 * CUE))H_2O$$

Relative degree of reduction: $\gamma_D = 4 \cdot n_C + 1 \cdot n_H - 2 \cdot n_O + 6 \cdot n_S + 5 \cdot n_P$

Glucose: $\gamma_s = 4 * 6 + 1 * 12 - 2 * 6 = 24$, 1-mol glucose: $\gamma_s = \frac{24}{6} = 4$

Biomass-1: $\gamma_x = 4 + 1 * 1.6 - 2 * 0.5 = 4.6$

Biomass-2: $\gamma_x = 4 + 1 * 1.571 - 2 * 0.429 = 4.713$

Redox balance: $\gamma_S^C + Y_{O/S} \cdot -4 + CUE \cdot n_N^X \cdot \gamma_N = CUE \cdot \gamma_X$

Amount of oxygen: $Y_{O/S} = \frac{CUE * \gamma_X - n_N^X \cdot \gamma_N \cdot CUE - \gamma_S^C}{-4} = \frac{\gamma_S^C - (\gamma_X - n_N^X \cdot \gamma_N)CUE}{4}$

Reaction heat: $\Delta H_r = (-455) * Y_{\frac{O}{S}} = (-455) * \frac{\gamma_S^C - (\gamma_X - n_N^X \cdot \gamma_N)CUE}{4}$

Calorespirometric ratio: $\mathrm{CR} = \frac{(-455) \cdot (\gamma_S^C - (\gamma_X - n_N^X \cdot \gamma_N)CUE)}{4 \cdot (1 - CUE)}$

Carbon use efficiency: $CUE = \frac{\frac{4\mathrm{CR}}{-455} - \gamma_S^C}{\frac{4CR}{-455} + (n_N^X \cdot \gamma_N - \gamma_X)}$

2 Supplementary Figures and Tables

2.1 Specific growth rate

Heat flow data were fitted via "growthrates" package in R studio and coefficients were calculated afterwards (https://cran.r-project.org/web/packages/growthrates/growthrates.pdf). This package roughly distinguishes between lag phase and exponential growth phase of soil systems and follows the procedure described by (here add the reference). Data were smoothed by re-exporting data points every 20 minutes considering the signal noise originating from device itself. μ_{max} stands for the apparent specific maximum growth rate for exponential growth phase. P_0 is the starting point of exponential growth rate and P_0_lm is the intersection of the fit with the abscissa. The abscissa of the intersection point between the heat flow curve and $P=P_0$ is estimated as the lag phase duration.

In this package, the derivates $\Delta P/\Delta t$ was calculated for each data point and this method seeks for the steepest slope which is considered as the specific growth rate. All other adjacent datasets that have more than 95% of the maximum slope are also included in the exponential growth datasets. A linear model was fitted to the log-transformed dataset which gave the information about exponential growth phase.

$$P = e^{\mu_{max} * (t - t_{lag})} + P_0 \quad (2)$$

2.1.1 Specific growth rate (opening and closing)

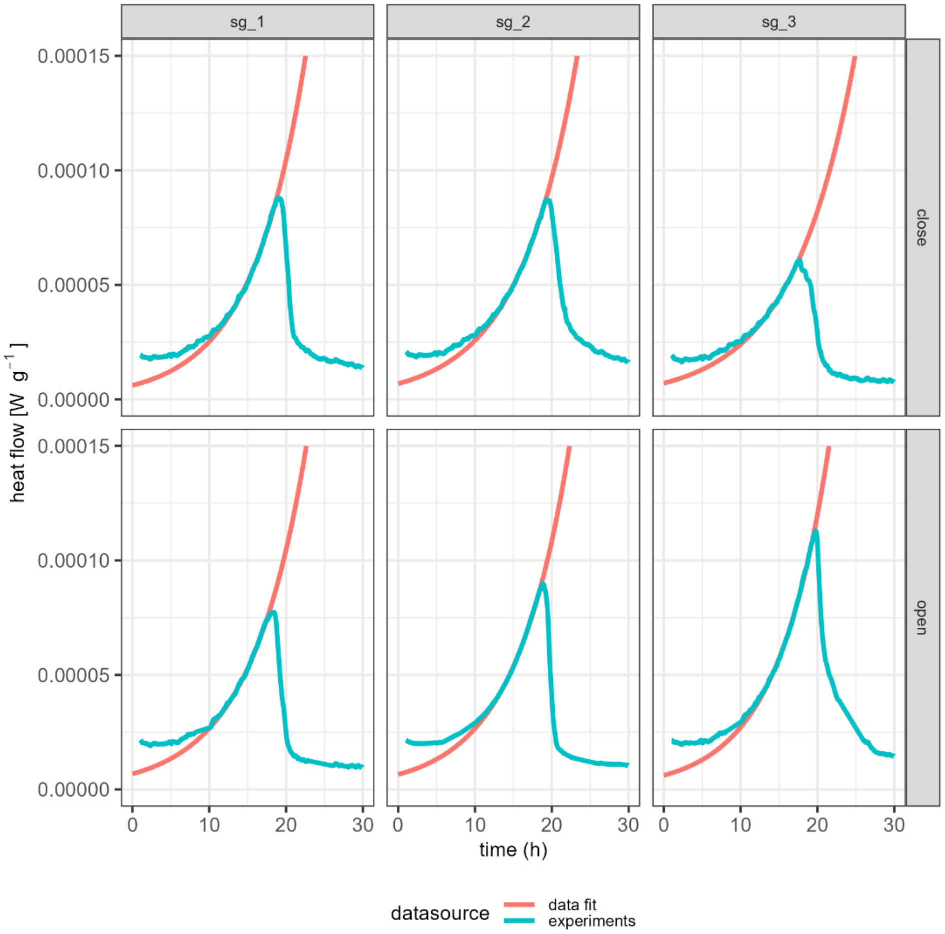

Figure 1 Exponential growth phase data fitting for open and close conditions

Triplicates for soil amended with glucose for closed ampoules and ampoules which are regularly aerated were set up and performed with TAM III. Figure 1 illustrates the fitted results with heat flow data. Opening the ampoules between the experiments resulted in disturbance for around 5 minutes due to immediate temperature changes. The starting and ending point of disturbances were selected and cut out. The resulting gaps were closed by interpolation between the signal. The red lines in Figure 1 were plotted according to the fitted parameter and the equation:

$$P = e^{\mu_{max} * (t - t_{lag})} + P_0$$

Time scales were limited to 30 hours considering tiny heat release during stationary phase.

Table 1 Coefficients for fitted data (open and close)

channel	P_0 [10^{-5} W g^{-1}]	P_0_lm [10^{-6} W g^{-1}]	μ_{app} [h^{-1}]	t_{lag} [h]	treatment	state
sg1	1.843	6.094	0.1421	7.789	soil+glucose	close
sg2	1.996	6.842	0.1324	8.084	soil+glucose	close
sg3	1.890	7.054	0.1228	8.022	soil+glucose	close
Mean Value	1.910	6.663	0.1325	7.965	soil+glucose	close
Standard error	0.0451	0.2912	0.0056	0.0899	soil+glucose	close
sg1	2.139	6.801	0.1366	8.3870	soil+glucose	open
sg2	2.083	6.532	0.1405	8.2582	soil+glucose	open
sg3	2.119	6.166	0.1485	8.3120	soil+glucose	open
Mean Value	2.114	6.500	0.1419	8.3190	soil+glucose	open
Standard error	0.0163	0.1838	0.0035	0.0373	soil+glucose	open

Mean value and standard error were summarized in Table 1. For close ampoules, $\mu_{max} = 0.1325 \pm 0.0056\ h^{-1}$. While for regularly open ampoules, $\mu_{max} = 0.1419 \pm 0.0035 h^{-1}$

2.2 Specific growth rate (different devices)

Apart from accumulated heat, specific growth rate was also fitted and compared between three devices with different detection limitation along with ampoule size.

2.2.1 Specific growth rate (TAM Air)

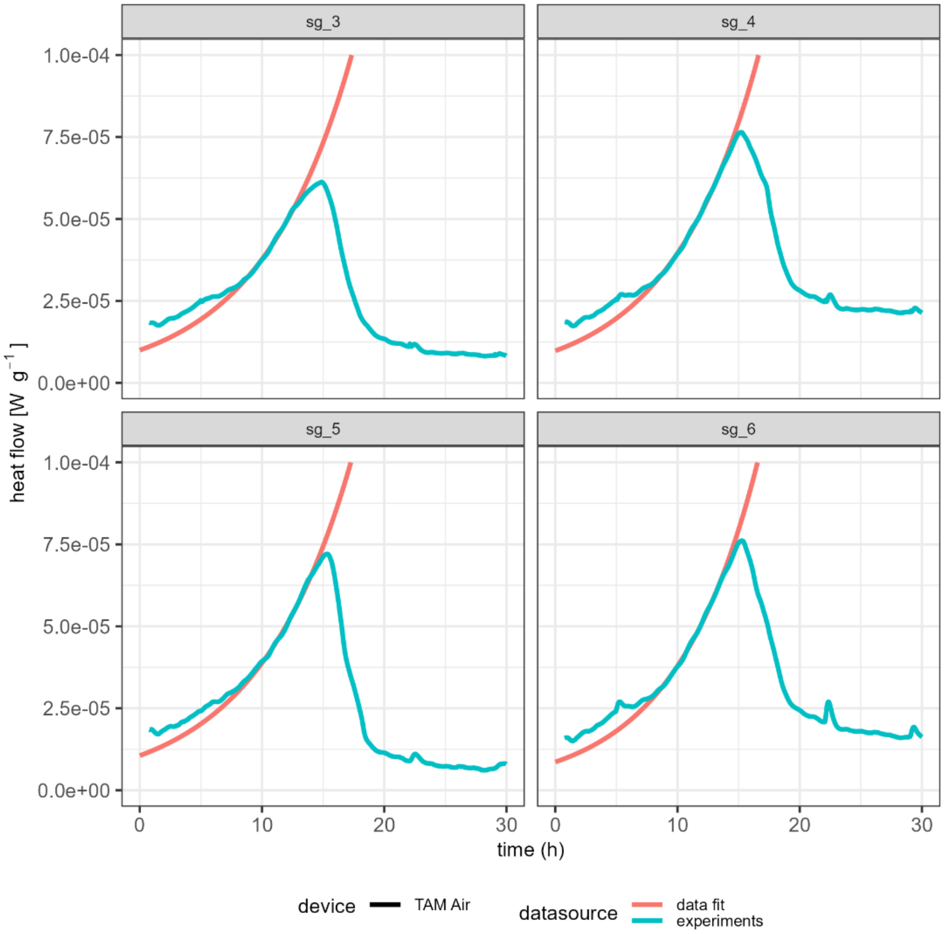

Figure 2 Exponential growth phase data fitting for TAM Air

Four replicates for soil amended with glucose in closed ampoules -were set up TAM Air. Figure 2 presents the results of fitted parameters for TAM Air. the fitted results with heat flow data. As shown in table 2, the mean $\mu_{max} = 0.1377 \pm 0.0040 h^{-1}$.

Table 2 Coefficients for fitted data (TAM Air)

channel	P_0 [10^{-5} W g^{-1}]	P_0_lm [10^{-6} W g^{-1}]	μ_{max} [h^{-1}]	t_{lag} [h]
sg3	1.832	9.993	0.1328	4.5614
sg4	1.859	9.801	0.1396	4.5837
sg5	1.855	10.565	0.1301	4.3245
sg6	1.590	8.626	0.1481	4.1303
Mean Value	1.784	9.746	0.1377	4.4000
Standard error	0.0648	0.4072	0.0040	0.1073

2.2.2 Specific growth rate (TAM III)

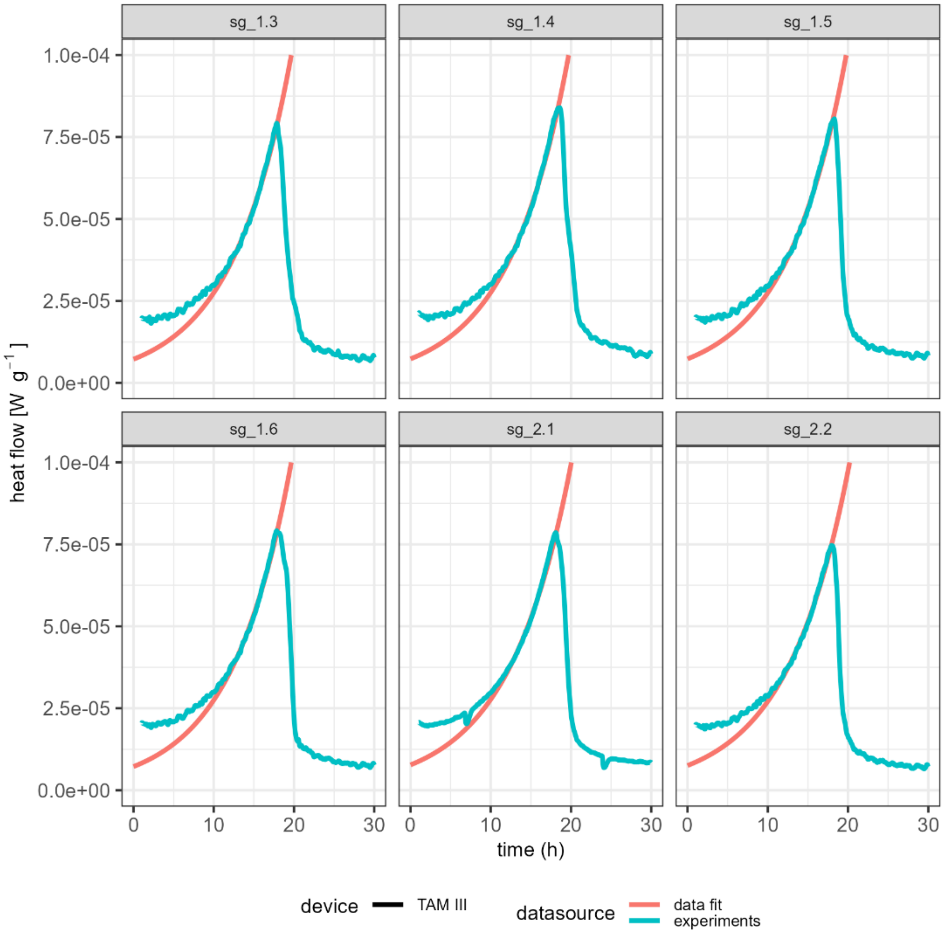

Figure 3 Exponential growth phase data fitting for TAM III

Six replicates for soil amended with glucose under close were set up with TAM III. Similar procedures were conducted doe heat flow data generated from TAM III. As shown in Table 3, the mean $\mu_{max} = 0.1312 \pm 0.0011h^{-1}$.

Table 3 Coefficients for fitted data (TAM III)

channel	P_0 [10^{-5} W g^{-1}]	P_0_lm [10^{-6} W g^{-1}]	μ_{app} [h^{-1}]	t_{lag} [h]
sg1.3	1.953	7.237	0.1335	7.436
sg1.4	2.139	7.333	0.1326	8.074
sg1.5	2.013	7.339	0.1321	7.639
sg1.6	2.062	7.227	0.1336	7.848
sg2.1	2.067	7.737	0.1277	7.697
sg2.2	2.010	7.543	0.1279	7.661
Mean Value	2.041	7.403	0.1312	7.726
Standard error	0.0259	0.0814	0.0011	0.0881

2.2.3 Specific growth rate (Mc-Cal/100P)

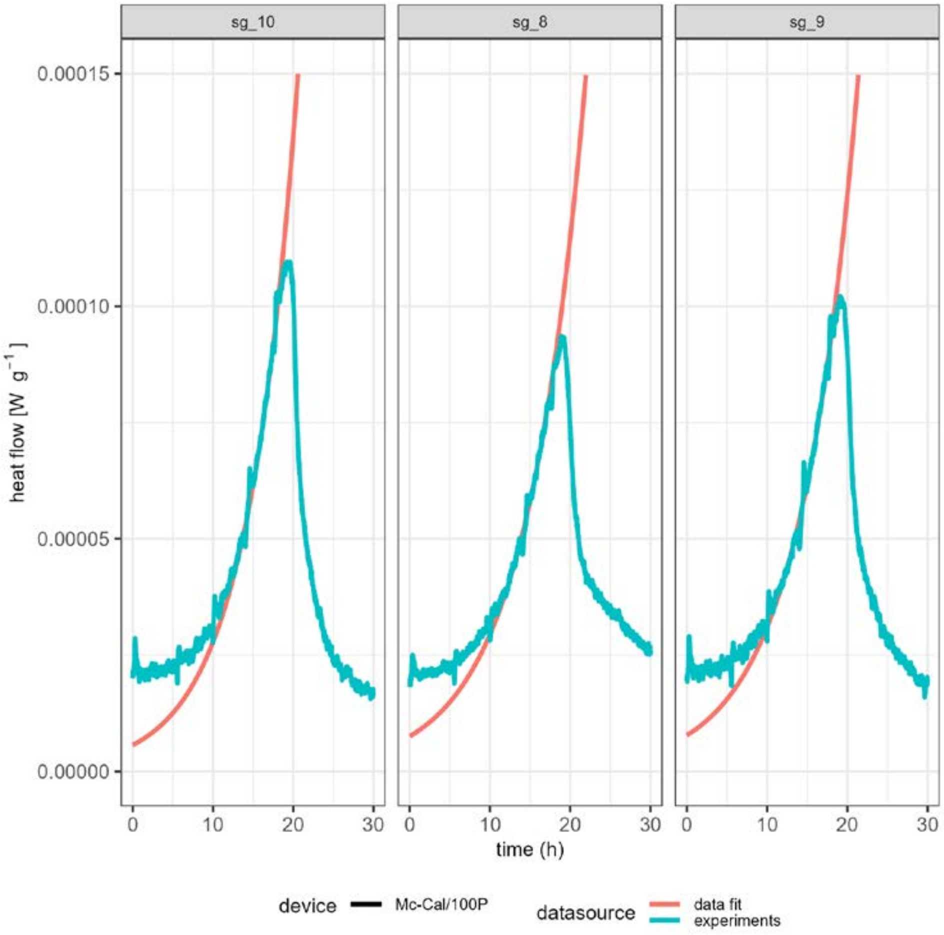

Figure 4 Exponential growth phase data fitting for Mc-Cal/100P

Triplicates for soil amended with glucose in closed ampoules were set up with Mc-Cal/100P. The mean $\mu_{max} = 0.1445 \pm 0.0073h^{-1}$ for Mc-Cal/100P.

Table 4 Coefficients for fitted data (Mc-Cal/100P)

channel	P_0 [10^{-5} W g^{-1}]	P_0_lm [10^{-6} W g^{-1}]	μ_{max} [h^{-1}]	t_{lag} [h]
sg8	2.787	5.666	0.1590	10.0187
sg9	2.466	7.559	0.1361	8.6915
sg10	2.906	7.768	0.1384	9.5334
Mean Value	2.720	6.998	0.1445	9.4145
Standard error	0.1313	0.6686	0.0073	0.3877
